# Joint factor analysis and approximate equipercentile linking of common trait health anxiety measures: a cross-sectional study of the 14-, 18- and 64-item health anxiety inventory, the illness attitude scale, and the 14-item Whiteley Index

**DOI:** 10.1186/s12888-023-05151-7

**Published:** 2023-09-06

**Authors:** Erland Axelsson, Susanna Österman, Erik Hedman-Lagerlöf

**Affiliations:** 1 Division of Family Medicine and Primary Care, Department of Neurobiology, Care Sciences and Society, Karolinska Institutet, Stockholm, Sweden; 2 Liljeholmen Primary Health Care Center, Region Stockholm, Liljeholmstorget 7, Stockholm, SE-117 94 Sweden; 3 Academic Primary Health Care Center, Region Stockholm, Stockholm, Sweden; 4 Centre for Psychiatry Research, Department of Clinical Neuroscience, Karolinska Institutet, Stockholm, Sweden; 5 Division of Psychology, Department of Clinical Neuroscience, Karolinska Institutet, Stockholm, Sweden; 6 Gustavsberg Primary Health Care Center, Region Stockholm, Stockholm, Sweden

**Keywords:** Health anxiety, Hypochondriasis, Illness anxiety disorder, Linking, Somatic symptom disorder

## Abstract

**Background:**

Research on health anxiety has bloomed in recent years, but summaries of the literature are complicated by the use of dissimilar self-report questionnaires. Furthermore, these instruments have rarely been administered in parallel, and especially not in clinical samples. In this study, we aimed to investigate the relationship between five widespread health anxiety measures, and to draft guidelines for the conversion of different sum scores.

**Methods:**

Clinical trial participants with principal pathological health anxiety (n = 335) and a sample of healthy volunteers (n = 88) completed the 14-item Whiteley Index (WI-14), the Illness Attitude Scale (IAS), and the 14-, 18-, and 64-item Health Anxiety Inventory (the HAI-64, HAI-18, and HAI-14). Cross-sectional data from all participants were pooled (N = 423) and we conducted a joint factor analysis and approximate equipercentile linking of the WI-14, IAS, HAI-64, HAI-18, and HAI-14.

**Results:**

Inter-scale correlations were high (rs ≥ 0.90 and ≥ 0.88 in adjusted analyses), and the scree plot of the joint factor analysis spoke for a unifactorial solution where 89/105 items (85%) had loadings ≥ 0.40. Most items at the core of this broad trait health anxiety factor pertained to the worry about health, the fear of having or developing a serious disease, and to some extent bodily preoccupation. We present a cross-walk table of observed equipercentile linked sum scores.

**Conclusions:**

This study speaks clearly in favor of the WI-14, IAS, HAI-64, HAI-18, and HAI-14 all tapping into the same trait health anxiety construct, the core of which appears to concern the worry about health, the fear of having or developing a serious disease, and to some extent bodily preoccupation. Based on recently reported cut-offs for the HAI-14, a reasonable cutoff for pathological health anxiety in a psychiatric setting probably lies around 7–8 on the WI-14, 52–53 on the IAS, 82–83 on the HAI-64, and 26–27 on the HAI-18.

**Trial registration:**

ClinicalTrials.gov NCT01966705, NCT02314065.

**Supplementary Information:**

The online version contains supplementary material available at 10.1186/s12888-023-05151-7.

## Introduction

Health anxiety is a multifaceted psychological trait that is primarily characterized by a fear of, or preoccupation with, having or developing a serious health condition such as terminal cancer, a severe cardiovascular disease, or a progressive neurological disorder [1]. Higher levels of trait health anxiety commonly imply increased anxiety sensitivity and intolerance of uncertainty [2], increased bodily preoccupation [3], increased frequency and intensity of behaviors aimed at reducing health anxiety in the short term [4], and a more pronounced fear of death [5]. In the general population, trait health anxiety varies on a continuum, from benign levels of concern about health, to pathological health anxiety that is both recurrent and excessive [6–9]. Based on its clinical characteristics, pathological health anxiety can be considered a *de facto* anxiety or perhaps obsessive-compulsive spectrum disorder [2, 10–14].

Over the past decades, research focusing on health anxiety has been growing rapidly for several reasons; one being the relative success of the cognitive-behavioral framework in guiding treatment efforts and promoting interest in experimental work [11, 15]. Other factors sparking an interest in health anxiety include a rise in work focusing on the role of information technology in exacerbating health concerns [16], emergent discrepancies in widespread diagnostic taxonomies [14, 17], and an increased interest in the role of psychological factors in somatic disease including covid-19 [18, 19]. However, an obstacle to the interpretation of this increasingly diverse research field is that a large number of different trait health anxiety measures are in circulation, and that these are not easily compared. Thus, it is often difficult to determine whether participants of different studies suffer from similar levels of trait health anxiety, and if so, whether these levels are indicative of pathological health anxiety.

Self-report measures of trait health anxiety have existed since the mid-1960s. The 14-item Whiteley Index with dichotomous (“yes”/”no”) items (WI-14) was developed based on hospital staff definitions of “hypochondriasis” [20]. Its psychometric properties are usually found to be acceptable but not ideal, and revised versions with Likert-type items are now more common [21]. The Illness Attitude Scale (IAS; and sometimes referred to in the plural) was probably the most widely used measure of trait health anxiety from about the late 1980s to the early 2000s [22], and was developed on the basis of statements by patients who exhibited abnormal illness behavior or believed they had an undiagnosed disease [23]. Its psychometric properties are usually found to be good, though the factor structure is disputed [24]. The Health Anxiety Inventory [1] is perhaps the most widely used self-report measure of trait health anxiety today, and exists in many forms, the three most common probably being the 64-, 18-, and 14-item versions (HAI-64, HAI-18, and HAI-14). This questionnaire was developed to capture the cognitive and emotional components of DSM-IV hypochondriasis which was the prototypical pathological health anxiety diagnosis up until 2013. Common versions of the Health Anxiety Inventory are all widely believed to possess good to excellent psychometric properties [1, 25]. In a recent study, a cutoff of 22 on the HAI-14 was found to be appropriate for identifying patients with pathological health anxiety in the psychiatric setting [26]. When the respondent is known to suffer from pathological health anxiety, a score of 28 or higher is indicative of moderate symptoms, and 33 of substantial symptoms [26]. In summary, the WI-14, IAS, HAI-64, HAI-18 and HAI-14 are all examples of widespread measures of trait health anxiety, but knowledge about how scores can be converted from one measure to another is lacking.

In this study, we aimed to investigate the relationship between the WI-14, IAS, HAI-64, HAI-18, and HAI-14 using a composite dataset derived from two clinical trials for pathological health anxiety and a sample of healthy volunteers. In a joint factor analysis, we aimed to test our hypothesis that the scales would be highly correlated and tap into the same latent trait health anxiety construct. Should such a broad latent trait health anxiety factor be present, we aimed to determine what cognitive, emotional, and behavioral characteristics that lie at the core of this factor. Furthermore, we intended to relate the sum scores of the WI-14, IAS, HAI-64, HAI-18, and HAI-14 by means of equipercentile linking. Using the resulting linking table, we intended to make use of recently developed guidelines for interpreting the HAI-14 [26] so as to draw conclusions about approximate cut-offs and guidelines for interpreting severity in terms of the WI-14, IAS, HAI-64, and HAI-18.

## Methods

### Design

This was a psychometric study based on cross-sectional data from a composite aduld sample (pooled N = 423) of 335 adult participants of two clinical trials of cognitive behavior therapy for pathological health anxiety [27, 28] and 88 healthy volunteers recruited via newspaper advertisements [29]. Notably, the two clinical trials included 336 participants but 1 was dropped from the present study due to missing WI-14 data. This study was a collaboration between Gustavsberg Primary Care Clinic and Karolinska Institutet, Stockholm, Sweden. All procedures were approved by the regional ethics review board of Stockholm (2013/375 − 31/5, 2014/1530-31/2), all participants gave informed consent to participate in research, and both clinical trials were preregistered at ClinicalTrials.gov (NCT01966705, NCT02314065).

### Procedure

All participants in the clinical trials exhibited a fear of, or preoccupation with, severe illness and met full criteria for a principal diagnosis of DSM-5 somatic symptom disorder or illness anxiety disorder as determined by a clinical psychologist aided by the Health Preoccupation Diagnostic Interview [HPDI; 30] and the Mini-International Neuropsychiatric Interview [MINI; 31]. The main exclusion criteria were a serious somatic condition, a substance use disorder, a psychotic disorder, a bipolar disorder, severe depression, and recurrent suicidal ideation. The healthy volunteers were assessed using the MINI and included only if found to be healthy. Prior to the eligibility interview, all 423 participants (both the clinical trial participants and the healthy volunteers), completed the self-report trait health anxiety measures as listed below.

### Outcomes

We administered the HAI-64, IAS, and WI-14 online in Swedish, using previously evaluated translations [1, 20, 23, 29]. Participants completed the questionnaires via their web browser, with black text on white background and radio buttons to mark responses. On the HAI-64, each of the 64 items renders a score of 0–3 and the respondent is encouraged to select one of four statements that best corresponds to their level of trait health anxiety (e.g., from *“I do not worry about my health”* to *“I spend most of my time worrying about my health”*). In this study, the HAI-64 had a theoretical range of 0-192 and was also rescored as the HAI-18 with a range of 0–54, and the HAI-14 with a range of 0–42, so as to enable approximate linking. The main difference, besides the number of items, between the HAI-14 on the one hand and the HAI-64 and HAI-18 on the other, is that the latter versions include a “negative consequences” subscale, which measures the perceived negative consequences of developing a serious disease. In this study, internal consistency was excellent for the sum scales of all versions of the Health Anxiety Inventory, i.e., the HAI-64 (α = 0.99), HAI-18 (α = 0.97), and HAI-14 (α = 0.97). On the IAS, 27 items are each scored 0–4, and responses indicate the frequency of various experiences pertaining to “worry about illness”, “concerns about pain”, “health habits”, “hypochondriacal beliefs”, “tanatophobia”, “disease phobia”, “bodily preoccupations”, “treatment experience”, and “effects of symptoms”, giving the sum score a theoretical range of 0-108. The internal consistency of the IAS in this study was excellent (α = 0.97). On the WI-14, each item is scored 0 (“*no*”) or 1 (“*yes*”), resulting in a theoretical range of 0–14. The internal consistency of the WI-14 in this study was excellent (α = 0.94).

### Statistical analysis

We conducted all statistical analyses in Stata 15.1. First, we validated that the trait health anxiety scales measured the latent construct, and were suitable for equipercentile linking. We calculated Pearson correlations and conducted a joint factor analysis of all (64 + 27 + 14 = 105) items, based on principal axis factoring with promax rotation. Considering that we expected factor loadings to be strong, factors to be few, and there to be many items per factor, we regarded the sample size of 423 as sufficient for this purpose [32, 33]. Because the HAI-64 is considerably longer than the IAS and WI-14, as a sensitivity analysis, we also conducted a secondary factor analysis that only included the items of the HAI-14, IAS, and WI-14. When we had established that all questionnaires tapped into the same latent trait health anxiety construct, we proceeded to equipercentile linking of the WI-14, IAS, HAI-64, HAI-18, and HAI-14. Equipercentile linking is a procedure whereby scores on scales measuring the same thing are linked by means of percentiles, so that scores on two scales are assumed to be equivalent if they correspond to the same percentile of each respective scale distribution. As is commonly done for discrete scales [34], we defined the percentile of each score as the percentage of participants scoring below that score, plus the percentage of participants having exactly that score divided by two. We linked sum scores in the ranges that were observed (represented in the sample), and based on presmoothed frequency distributions that allowed us to model all sum scores (including those not directly observed) within each such range. As a sensitivity analysis, we also report linked sum scores based on observed (non-smoothed) frequency distributions as supplementary material.

## Results

### Sample characteristics

The pathological health anxiety participants completed their questionnaires between December 4th 2013 and March 22nd 2014 (n = 131), and December 10th 2014 and March 15th 2017 (n = 204), in each respective clinical trial, and the healthy volunteers completed their questionnaires between March 19th 2014 and June 3rd 2014 (n = 88). A typical pathological health anxiety participant was a 38 years old (SD = 12) female (240/335, 72%) with a tertiary education (259/335, 77%) who scored in the moderate pathological range of trait health anxiety (Table 1). In this subsample, 64/335 (19%) had comorbid depression and 207/335 (62%) met full criteria for at least one comorbid anxiety disorder, post-traumatic stress disorder, or obsessive-compulsive disorder. A typical healthy volunteer was a 49 years old (SD = 18) female (59/88, 67%) with a tertiary education (80/88, 91%) who scored low on trait health anxiety (Table 1). None of the healthy volunteers was found to meet criteria for a psychiatric disorder. In the pooled sample (N = 423), the distribution of trait health anxiety scores covered most but not all theoretically possible sum scores (the WI-14 being the exception where all scores, 0–14, were observed; see the supplementary material for details).

**Table 1:** Sample trait health anxiety sum scores

		Pathological health anxiety (*n* = 335)	Healthy volunteers (*n* = 88)	Total (*N* = 423)
HAI-14	M (SD), range	29.3 (4.7), 16–41	6.4 (3.8), 0–21	24.5 (10.3), 0–41
	Median (IQR)	29 (32 − 27)	5 (8 − 4)	28 (31 − 21)
HAI-18	M (SD), range	35.5 (6.2), 19–53	8.1 (4.5), 0–28	29.8 (12.6), 0–53
	Median (IQR)	36 (40 − 31)	7 (11 − 5)	34 (39 − 26)
HAI-64	M (SD), range	111.8 (20.4), 54–174	30.4 (13.3), 9–88	94.9 (38.2), 9-174
	Median (IQR)	112 (126 − 98)	28.5 (37.5–21.5)	104 (121 − 80)
IAS	M (SD), range	70.8 (12.3), 41–103	21.0 (8.6), 5–55	60.5 (23.3), 5-103
	Median (IQR)	71 (79 − 63)	20 (26.5–15)	67 (76 − 52)
WI-14	M (SD), range	10.7 (2.0), 5–14	1.1 (1.2), 0–7	8.7 (4.3), 0–14
	Median (IQR)	11 (12 − 9)	1 (2 − 0)	10 (12 − 7)

*Note*. “Range” refers to observed values as opposed to theoretical ranges. HAI = Health Anxiety Inventory (14, 18, and 64-item version as indicated); IAS = Illness Attitude Scale; IQR = interquartile range; WI-14 = 14-item Whiteley Index with dichotomous (“yes”/”no”) items

### Joint factor analysis and feasibility of equipercentile linking

In the pooled sample, Pearson correlations between the trait health anxiety measures were all high (≥ 0.90 and ≥ 0.88 in adjusted analyses; Table 2) and the Kaiser-Meyer-Olkin (KMO) statistic was indicative of adequate sampling (0.98). Joint factor analysis of the HAI-64, IAS, and WI-14 pointed to one factor being clearly dominant, as is illustrated in the scree plot in Figure S1. All three scales contributed with items that received factor loadings in at least the high 0.80s. The common theme of these core items appeared to be worry about health and the fear of having or developing a serious disease, notable examples being HAI-64 #8 (i.e., HAI-14 and HAI-18 #5: *“I am […] afraid that I have a serious illness”*), IAS #1 (*“Do you worry about your health?“*), and WI-14 #1 and #4 (*“Do you often worry about the possibility that you have got a serious illness?“*, *“Do you worry a lot about your health?“*). Weak items were primarily found in the HAI-64 Negative Consequences (NC) subset (i.e., #48 onwards), and were primarily items that concerned social interaction in the case of serious illness (Tables S1 and S2). When two factors were retained in the analysis, most of the 17 NC items mapped onto a relatively weak second factor (Tables S3 and S4). Overall, however, this factor analysis indicated that the WI-14, IAS, HAI-64, HAI-18, and HAI-14 tap into one and the same strong trait health anxiety factor as their primary source of variance, and results were similar when the factor analysis included the items of the WI-14, IAS, and HAI-14 only (Figure S2, Tables S5 and S6).

**Table 2:** Pearson correlation matrix of trait health anxiety measures

	HAI-14	HAI-18	HAI-64	IAS	WI-14
HAI-14	-				
HAI-18	0.99	-			
HAI-64	0.97	0.99	-		
IAS	0.94	0.93	0.94	-	
WI-14	0.92	0.91	0.90	0.92	-
HAI-64 minus HAI-14	0.95	0.97	1.00	0.92	0.88
HAI-64 minus HAI-18	0.96	0.97	1.00	0.93	0.89

*Note*. All values are significant (P < 0.0001), though note that because the HAI-64 was rescored as the HAI-18 and HAI-14, item scores were often identical per definition and the correlations between these three measures are therefore inflated. Adjusted correlations with the HAI-64 minus the HAI-18 and HAI-14 are therefore included. HAI = Health Anxiety Inventory (14, 18, and 64-item version as indicated); IAS = Illness Attitude Scale; WI-14 = 14-item Whiteley Index with dichotomous (“yes”/”no”) items

### Equipercentile linking of trait health anxiety sum scores

Because all trait health anxiety scales appeared to be closely associated, we proceeded to equipercentile linking of their sum scores. Table 3 can be used for approximate linking of sum scores on the WI-14, IAS, HAI-64, HAI-18, and HAI-14. With the help of Table 3, each trait health anxiety score that was observed in the present study can be linked to its percentile, which in turn can be linked to a score on another trait health anxiety scale. Thus, for example, a score of 22 on the HAI-14 corresponds to approximately 26–27 on the HAI-18. Linking based on non-presmoothed frequency distributions resulted in relatively similar outcomes.

**Table 3:** Cross-walk table of equipercentile linked scores on trait health anxiety measures

Percentile	HAI-14	HAI-18	HAI-64	IAS	WI-14		Percentile	HAI-14	HAI-18	HAI-64	IAS	WI-14
0.045		0					29.339				55	
0.060				5			30.119			87		
0.119			9				30.664				56	
0.133	0						30.665		28			
0.212				6			30.986			88		
0.291		1					31.592	24				
0.364			10				31.897			89		
0.441				7			32.085				57	
0.602	1						32.502					8
0.630			11				32.851			90		
0.754				8			33.333		29			
0.916		2					33.611				58	
0.924			12				33.850			91		
1.142				9			34.772	25				
1.251			13				34.890			92		
1.597				10			35.243				59	
1.614			14				35.968			93		
1.622	2						36.343		30			
1.967		3					36.971				60	
2.014			15				37.080			94		
2.116				11			38.220			95		
2.449			16				38.781				61	
2.710				12			39.097	26				
2.917			17				39.386			96		
3.385		4					39.719		31			
3.389				13			40.255					9
3.396	3						40.572			97		
3.412			18				40.657				62	
3.553					0		41.777			98		
3.931			19				42.594				63	
4.156				14			43.000			99		
4.471			20				43.472		32			
5.006				15			44.240			100		
5.028			21				44.594				64	
5.095		5					44.648	27				
5.601			22				45.498			101		
5.803	4						46.667				65	
5.926				16			46.776			102		
6.187			23				47.601		33			
6.784			24				48.076			103		
6.895				17			48.819				66	
6.989		6					49.400			104		
7.390			25				49.498					10
7.884				18			50.751			105		
8.003			26				51.055				67	
8.437	5						51.110	28				
8.619			27				52.103		34			
8.874				19			52.128			106		
8.908		7					53.364				68	
9.236			28				53.528			107		
9.850			29				54.949			108		
9.853				20			55.734				69	
10.455			30				56.386			109		
10.465					1		56.905		35			
10.712		8					57.834			110		
10.810				21			58.135	29				
10.879	6						58.151				70	
11.050			31				59.287			111		
11.629			32				60.599				71	
11.733				22			60.739			112		
12.190			33				61.750					11
12.348		9					61.853		36			
12.614				23			62.188			113		
12.728			34				63.059				72	
12.919	7						63.633			114		
13.244			35				65.075			115		
13.443				24			65.209	30				
13.735			36				65.516				73	
13.817		10					66.512			116		
14.203			37				66.784		37			
14.208				25			67.942			117		
14.629	8						67.959				74	
14.650			38				69.363			118		
14.905				26			70.372				75	
15.075			39				70.769			119		
15.109		11					71.581		38			
15.481			40				71.782	31				
15.537				27			72.155			120		
15.868			41				72.737				76	
16.082					2		73.516			121		
16.113				28			74.848			122		
16.124	9						75.041				77	
16.222		12					76.132		39			
16.235			42				76.146			123		
16.584			43				76.974					12
16.641				29			77.274				78	
16.913			44				77.406			124		
17.131				30			77.543	32				
17.175		13					78.628			125		
17.223			45				79.420				79	
17.329	10						79.808			126		
17.515			46				80.299		40			
17.590				31			80.947			127		
17.790			47				81.456				80	
17.984		14					82.041			128		
18.019				32			82.519	33				
18.047			48				83.092			129		
18.187	11						83.364				81	
18.286			49				84.008		41			
18.413				33			84.098			130		
18.509			50				85.064			131		
18.642		15					85.131				82	
18.716			51				85.993			132		
18.764				34			86.754				83	
18.790	12						86.886			133		
18.909			52				86.920	34				
19.075				35			87.275		42			
19.089			53				87.748			134		
19.144		16					88.233				84	
19.223	13						88.578			135		
19.259			54				89.375			136		
19.321					3		89.576				85	
19.348				36			90.120		43			
19.421			55				90.139			137		
19.524		17					90.776	35				
19.576			56				90.790				86	
19.583	14						90.870			138		
19.590				37			91.181					13
19.726			57				91.568			139		
19.810				38			91.881				87	
19.838		18					92.233			140		
19.872			58				92.526		44			
19.947	15						92.860				88	
20.012			59				92.865			141		
20.024				39			93.463			142		
20.147		19					93.745				89	
20.148			60				93.866	36				
20.246				40			94.026			143		
20.278			61				94.499		45			
20.327	16						94.551				90	
20.405			62				94.555			144		
20.486				41			95.047			145		
20.502		20					95.281				91	
20.530			63				95.505			146		
20.634					4		95.930			147		
20.656			64				95.939				92	
20.753				42			96.026	37				
20.759	17						96.083		46			
20.786			65				96.323			148		
20.924			66				96.532				93	
20.947		21					96.686			149		
21.049				43			97.020			150		
21.074			67				97.068				94	
21.240			68				97.319		47			
21.311	18						97.328			151		
21.335					5		97.428	38				
21.377				44			97.552				95	
21.424			69				97.609			152		
21.520		22					97.866			153		
21.628			70				97.990				96	
21.735				45			98.099			154		
21.854			71				98.212		48			
22.091	19						98.308			155		
22.103			72				98.389				97	
22.129				46			98.412	39				
22.275		23					98.494			156		
22.377			73				98.652					14
22.566				47			98.657			157		
22.678			74				98.750				98	
22.719					6		98.799			158		
23.008			75				98.800		49			
23.060				48			98.921			159		
23.260	20						99.027			160		
23.283		24					99.066				99	
23.370			76				99.118			161		
23.628				49			99.169	40				
23.769			77				99.171		50			
24.205			78				99.198			162		
24.291				50			99.268			163		
24.608		25					99.331				100	
24.683			79				99.332			164		
24.916	21						99.391			165		
25.067				51			99.414		51			
25.204			80				99.448			166		
25.772			81				99.503			167		
25.964				52			99.547				101	
26.288		26					99.558			168		
26.308					7		99.613			169		
26.388			82				99.620		52			
26.904	22						99.668			170		
26.981				53			99.727			171		
27.051			83				99.729				102	
27.758			84				99.749	41				
28.109				54			99.791			172		
28.317		27					99.862		53			
28.506			85				99.865			173		
29.082	23						99.907				103	
29.293			86				99.952			174		

*Note.* Linking was based on presmoothed frequency distributions. Using this table, each trait health anxiety score can be linked to a percentile, which in turn can be linked to a score on another trait health anxiety scale. Thus, for example, a score of 22 on the HAI-14 corresponds to about 26–27 on the HAI-18. HAI = Health Anxiety Inventory (14, 18, and 64-item version as indicated); IAS = Illness Attitude Scale; WI-14 = 14-item Whiteley Index with dichotomous (“yes”/”no”) items

## Discussion

This study was an unusual attempt at a joint analysis of five common trait health anxiety self-report questionnaires. We found that the WI-14, IAS, HAI-64, HAI-18, and HAI-14 all loaded heavily on the same broad latent trait health anxiety factor, as illustrated by the fact that out of 105 items in total, 35 had loadings ≥ 0.80 and 89 had loadings ≥ 0.40. A strength of the present study is that all non-healthy participants had pathological health anxiety as opposed to other primary psychopathologies, so that a clear gradient in trait health anxiety, without substantial interference of partially overlapping constructs such as somatic disease and panic disorder symptoms, could be modelled for the purpose of factor analysis and the linking of sum scores. The strong unifactorial solution seen in this study speaks in favor of the linking of sum scores derived from the WI-14, IAS, HAI-64, HAI-18, and HAI-14.

Based on estimates derived from the cross-walk table (Table 3), an HAI-14 cutoff of 22 to screen for pathological health anxiety in the psychiatric setting [26] corresponds to a score of ca. 26–27 on the HAI-18, 82–83 on the HAI-64, 52–53 on the IAS, and 7–8 on the WI-14. These tentative cutoffs are slightly higher than those previously reported [35], most probably because the present estimates are derived from an analysis where consecutive psychiatric patients constituted the reference group [26] whereas previous estimates were derived from a study that employed a more pragmatic reference group [35]. In respondents with confirmed pathological health anxiety, based on the recent suggestion that scores below 28 on the HAI-14 are indicative of mild symptoms [26], the same could be said of scores below ca. 33–34 on the HAI-18, 105–106 on the HAI-64, 67–68 on the IAS, and 10–11 on the WI-14. Similar to scores of at least 33 on the HAI-14 [26], symptoms are probably to be regarded substantial even within the clinical range if the respondent scores at least ca. 40–41 on the HAI-18, 128–129 on the HAI-64, 80–81 on the IAS, or 12–13 on the WI-14. These approximate conversions and rule of thumb guidelines for the interpretation of scores derived from common trait health anxiety scales could be of use both in research and the clinic.

In this study, items that pertained to worrying about health and fearing the prospect of having or developing a serious disease were at the heart of the trait health anxiety construct. One implication of this finding is that, should a minimal set of questions be used for the purpose of identifying individuals with pathological health anxiety, for example by the general practitioner or as part of a screening procedure, these questions should ideally focus on worrying about health and the fear of having or developing a serious disease. Thus, for example, it would probably be more fruitful to ask *“Would you say that you worry a lot about your health, and the possibility of having a serious disease?”* than to ask about repeated doctor shopping or other aspects of the trait health anxiety construct. Importantly, this view of health worries and the fear of disease as the core of the trait health anxiety construct contrasts with certain widespread conceptualizations of pathological health anxiety, such as the ICD-11 hypochondriasis diagnosis, which focuses more on the presence of excessive health-related behaviors. Results for bodily preoccupation were mixed, and our impression is that items tapping into worry such as IAS #20 (*“When you notice a sensation in your body, do you find it difficult to think of something else?”*, 0.90–0.94) had substantial factor loadings whereas items that conceived of bodily preoccupation in terms of perceptual changes or “being aware” of the body only had slightly weaker or even modest loadings (e.g., HAI-64 #3: *“I am constantly aware of bodily sensations or changes”*, 0.74–0.75; WI-14 #3: *“Do you find that you are often aware of various things happening in your body?”*, 0.53–0.58). Items pertaining to the fear of death (IAS #13–14) showed mixed results in the 0.59–0.78 range, thus only partly corroborating the common view that the fear of death is an integral component of pathological health anxiety [5, 36]. Interestingly, not responding to medical reassurance (IAS #11, WI-14 #10), which used to be a criterion for hypochondriasis during the DSM-IV era, had relatively weak loadings (0.50–0.59). One possible explanation for this is that these medical reassurance items were phrased in a manner that led participants to reply based on whether they usually feel reassured in the very short term, as opposed to whether this reduction tends to persist for a longer period of time (*“When your doctor tells you that you have no physical disease to account for your symptoms, do you refuse to believe him?”*, *“Is it hard for you to believe the doctor when he tells you there is nothing for you to worry about?”*). The focus on this short time frame is unfortunate, considering that the cognitive-behavioral view of pathological health anxiety would contend that it is not so much the lack of a short-term reduction in anxiety that is characteristic of this clinical problem, but rather the reduced likelihood of a reduction in health anxiety being maintained over time [e.g., 37, 38]. Generally speaking, many items pertaining to overt behaviors such as symptom checking, reassurance seeking, and various avoidance behaviors were also in the lower range, highlighting that whereas high levels of trait health anxiety always imply an increased fear of or preoccupation with having or developing a serious disease, different individuals engage in different behaviors in the hope of reducing or managing this problem in the short term [4]. For example, an individual worrying about skin cancer may be more inclined to *“examine [his or her] body for disease”* (IAS #9), than an individual worrying about a severe congenital heart defect or pancreatic cancer. Interestingly, both being afraid of seeking health care (HAI-64 #22) and the inclination to seek healthcare (IAS #23 and #24) loaded on the same latent trait health anxiety trait which corroborates the previous finding that both patterns are common, and may even be found in the same individual and fluctuate over time [39]. Abnormal health behaviors are probably important for trait health anxiety, but measuring these using specific examples results in clear psychometric challenges.

We are aware of one previous study where more than one health anxiety scale was included in the same factor analysis [40]. In that study, 503 undergraduate students completed the HAI-18 and the Multidimensional Inventory of Hypochondriacal Traits [MIHT; 41]. When a second-order health anxiety factor was added, the MIHT Affective/Worry subfactor which focuses on worry about health and the fear about serious illness had the strongest factor loading (0.96), followed by a factor representing the first 14 items of the HAI-18 (i.e., the HAI-14; 0.83). Similar to the present study, the MIHT Behavioral/Reassurance subfactor had a modest factor loading of 0.55, and the Negative Consequences (NC) items of the HAI-18 did worse (0.51). Thus, on the whole, the outcome of the previous study was similar to the present one in the sense that the worry about health and the fear of serious illness was at the core of the trait health anxiety construct [40], which speaks for the validity of our findings. The fact that many of the HAI-64 NC items had relatively weak factor loadings on the broad trait health anxiety factor in both studies also has clinical implications in that this speaks for further use of the HAI-14 rather than the HAI-64 or HAI-18 if construct validity in the field as a whole is to be promoted. Simply put, the HAI-14 appears to focus on the core aspects of trait health anxiety.

This study had notable strengths. Several trait health anxiety questionnaires were administered in parallel which is unusual, especially in clinical samples. Furthermore, data could be derived from a combination of healthy volunteers and patients with pathological health anxiety which means that the full range of trait health anxiety scores were available for analysis. This study also had limitations. Participants were primarily self-referred, reported high average educational attainment, and were primarily female. This implies a threat that results may not generalize as well to populations that are not actively seeking treatment, that are less educated, and that are primarily male. Notably, relatively little is known about measurement invariance with regard to psychometric measures of health anxiety (for one of few noteworthy investigations, see MacSwain et al., 2009 [42]). This means that it is not clear to what degree the WI-14, IAS, HAI-64, HAI-18, and HAI-14 behave differently in psychometric terms for example as a function of demographic characteristics. Another limitation is that the HAI-14 and HAI-18 were scored from the corresponding items of the HAI-64 as opposed to administered separately, which may have affected the outcome due to intermediate items giving rise to framing and ordering effects [e.g., 43]. Because the HAI-64, IAS, and WI-14 were not administered in weighted or random order, ordering effects could also have affected the study overall, which highlights the preliminary nature of these findings. A limitation of this study is also that the full theoretical ranges of sum scores were not observed. Specifically, none of the participants had a score of 42 on the HAI-14, 54 on the HAI-18, 0–8 or 175–192 on the HAI-64, or 0–4 or 104–108 on the IAS. These sum score ranges were therefore not modelled. Last, we wish to highlight that several common trait health anxiety questionnaires were not included in the present study. For example, based on a decision taken around 2010 [44], in the research program from which these data were derived, we administered a dichotomous (“yes”/”no”) item version of the WI-14 as opposed to a Likert-version which is more common nowadays [21].

## Conclusion

This study indicates that the WI-14, IAS, HAI-64, HAI-18, and HAI-14 are all valid measures of the same trait health anxiety construct, the core of which appears to be the worry about health, the fear of having or developing a serious disease, and to some extent bodily preoccupation. Approximate linking guidelines enable clinicians and researchers to convert sum scores between questionnaires, and to determine how trait health anxiety levels compare over published studies. For the clinician, a take home message from this study could also be that it is probably more fruitful to identify patients with pathological health anxiety by asking them about health worries and the fear of serious disease (which appears to lies at the core of health anxiety) than to ask about specific behavioral patterns such as healthcare consumption or reassurance seeking which differ considerably between patients, and commonly change for the same patient over time.

### Electronic supplementary material

Below is the link to the electronic supplementary material.

Supplementary Material 1: Key output from joint factor analyses

Supplementary Material 2: Trait health anxiety sum score distributions

Supplementary Material 3: Sensitivity analysis: alternative linking based on non-smoothed frequency distributions

## Data Availability

The data analyzed during the current study are not publicly available due to the relevant Swedish and European Union data protection and privacy legislation, but are available from the corresponding author on reasonable request. Such requests will be considered on a case-by-case basis, so as to ensure that data and materials are stored, managed, and shared in accordance with the local policies of the sponsor and the relevant Swedish and European Union data protection and privacy legislation.
