# Supplementary material for: Joint factor analysis and approximate equipercentile linking of common trait health anxiety measures: a cross-sectional study of the 14-, 18- and 64-item health anxiety inventory, the illness attitude scale, and the 14-item Whiteley Index
Source: BMC Psychiatry. 2023 Sep 6;23:658. doi: 10.1186/s12888-023-05151-7 (PMC10483785; doi:10.1186/s12888-023-05151-7)
Supplement: Supplementary file 1 — Supplementary Material 1: Key output from joint factor analyses [file 12888_2023_5151_MOESM1_ESM.docx]

# Supplementary material:

# Key output from joint factor analyses

## Supplement of “Joint factor analysis and approximate equipercentile linking of common trait health anxiety measures: A cross-sectional study of the 14-, 18- and 64-item Health Anxiety Inventory, the Illness Attitude Scale, and the 14-item Whiteley Index”

### *Part I: Results from joint factor analysis of the 64-item Health Anxiety Inventory (HAI-64), the Illness Attitude Scale (IAS), and the 14-item Whiteley Index with yes/no items (WI-14)*


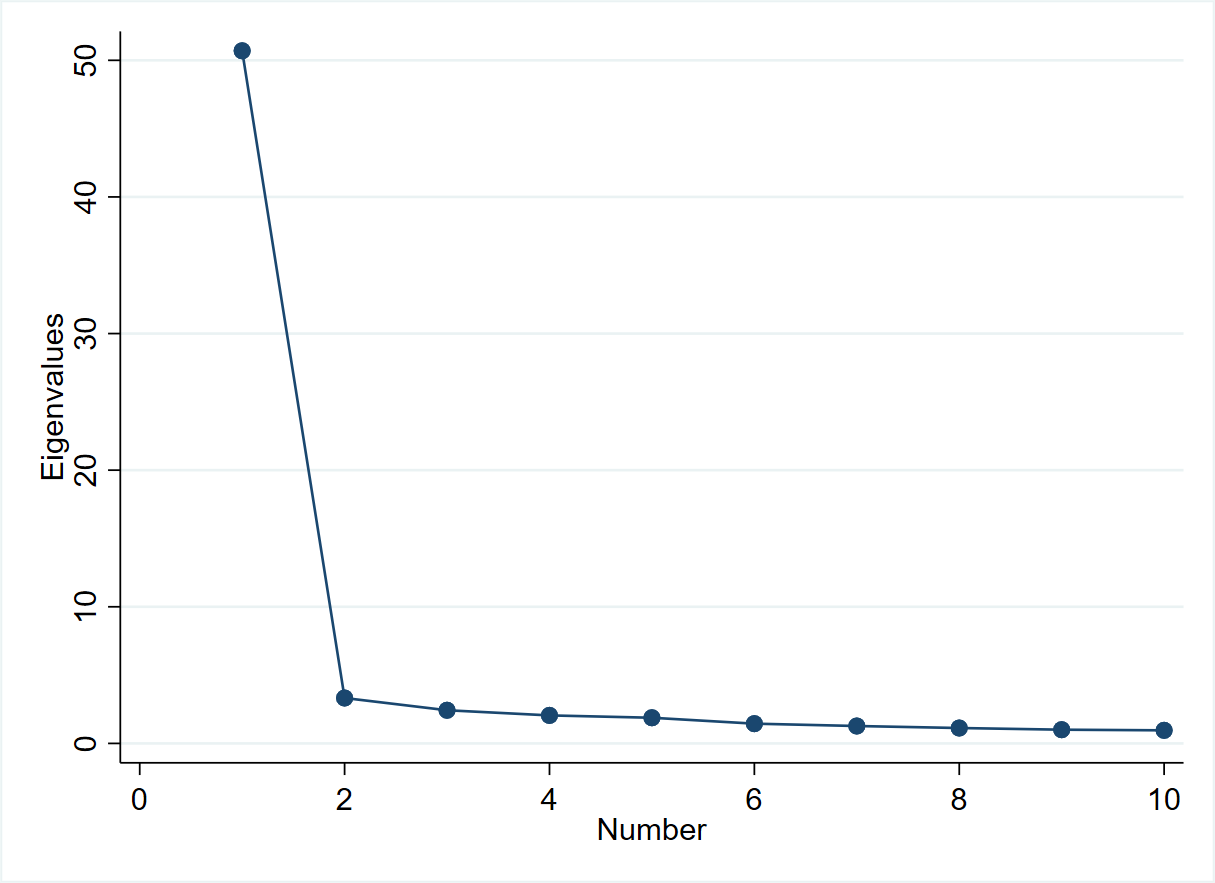


*Figure S1.* Scree plot of 10 highest eigenvalues.

*Table S1.* Factor loadings (pattern matrix) with one factor retained (67% variance explained), >0.90 marked *, ≥0.40 in bold text and ≥0.30 in italics, sorted according to scale.

| Item | Paraphrased text to facilitate interpretation | Loading |
| --- | --- | --- |
| HAI-64 #1 (SHAI) | *Time spent worrying about health* | **0.85** |
| HAI-64 #2 (SHAI) | *Awareness of aches and pains* | **0.74** |
| HAI-64 #3 (SHAI) | *Awareness of bodily sensations or changes* | **0.74** |
| HAI-64 #4 | *When bodily sensation, worries about it* | **0.86** |
| HAI-64 #5 (SHAI) | *Ability to resist thoughts of illness* | **0.85** |
| HAI-64 #6 | *How often worries about dying* | **0.82** |
| HAI-64 #7 | *When bodily sensation, thinks it is a sign of illness* | **0.88** |
| HAI-64 #8 (SHAI) | *Fear of having a serious illness* | **0.89** |
| HAI-64 #9 (SHAI) | *How often mental images of being ill* | **0.83** |
| HAI-64 #10 | *Perceived probability: serious illness in near future* | **0.83** |
| HAI-64 #11 | *If notices bodily sensation, how often checks on it* | **0.76** |
| HAI-64 #12 | *How realistic that the respondent is seriously ill* | **0.65** |
| HAI-64 #13 | *Avoidance of situations that trigger thoughts of death* | **0.62** |
| HAI-64 #14 | *If notices bodily sensation, how often focuses on it* | **0.82** |
| HAI-64 #15 | *How often examines body* | **0.62** |
| HAI-64 #16 (SHAI) | *Ability to take mind off thoughts about health* | **0.88** |
| HAI-64 #17 (SHAI) | *Not relieved if doctor says there is nothing wrong* | **0.83** |
| HAI-64 #18 (SHAI) | *If hears about illness, belief in having it* | **0.69** |
| HAI-64 #19 | *If notices sensations, tries to find the cause* | **0.63** |
| HAI-64 #20 | *How often health worries interfere with life* | **0.86** |
| HAI-64 #21 | *How often beliefs going to die soon* | **0.76** |
| HAI-64 #22 | *Afraid of visiting doctor because of health worries* | **0.60** |
| HAI-64 #23 | *Impact of worry about health on thinking about other* | **0.82** |
| HAI-64 #24 | *If notices sensation, how often attempt at distraction* | **0.69** |
| HAI-64 #25 | *How sensible is the idea of having serious illness* | **0.77** |
| HAI-64 #26 | *Previous illnesses mismanaged* | *0.33* |
| HAI-64 #27 (SHAI) | *Need to understand bodily sensations/changes* | **0.77** |
| HAI-64 #28 | *Avoids situations where illness is prominent* | **0.58** |
| HAI-64 #29 (SHAI) | *Perceived risk for developing a serious illness* | **0.79** |
| HAI-64 #30 | *How often images of dying or being dead* | **0.71** |
| HAI-64 #31 | *How often mentions unexplained bodily sensations* | **0.41** |
| HAI-64 #32 | *How often thinks about being seriously ill* | **0.74** |
| HAI-64 #33 | *Degree of perceived vulnerability to serious illness* | **0.80** |
| HAI-64 #34 | *If notices sensation, how often attempts reassurance* | **0.50** |
| HAI-64 #35 (SHAI) | *How often thinks that seriously ill* | **0.85** |
| HAI-64 #36 | *Perceived probability: becoming ill in next few weeks* | **0.78** |
| HAI-64 #37 | *How often afraid of developing a serious illness* | **0.87** |
| HAI-64 #38 | *Perceived probability: undiagnosed serious disease* | **0.70** |
| HAI-64 #39 | *Fear when thinking about developing serious illness* | **0.83** |
| HAI-64 #40 | *When in pain, thinks that sign of illness* | **0.82** |
| HAI-64 #41 | *How often feels as though going to die soon* | **0.65** |
| HAI-64 #42 | *If notices sensation, tries to get rid of it* | **0.44** |
| HAI-64 #43 (SHAI) | *If notices sensation, difficult think about other things* | **0.85** |
| HAI-64 #44 | *If hears about illness, worries about other illness* | **0.76** |
| HAI-64 #45 (SHAI) | *Family and friends believe worry to be excessive* | **0.83** |
| HAI-64 #46 | *GP’s belief in worry being excessive* | **0.73** |
| HAI-64 #47 | *Own belief in worry being excessive* | **0.76** |
| HAI-64 #48 (NC) | *If serious illness, would reduce quality of life* | **0.71** |
| HAI-64 #49 (NC) | *If serious illness, be very painful* | **0.56** |
| HAI-64 #50 (NC) | *If serious illness, would be fatal* | **0.67** |
| HAI-64 #51 (NC) | *If serious illness, prolonged suffering* | **0.64** |
| HAI-64 #52 (NC, SHAI) | *If serious illness, unable to enjoy life* | **0.72** |
| HAI-64 #53 (NC, SHAI) | *If serious illness, little belief in modern medicine* | **0.67** |
| HAI-64 #54 (NC) | *If serious illness, family/friends would express pity* | 0.23 |
| HAI-64 #55 (NC) | *If serious illness, reduced belief in own worth* | *0.36* |
| HAI-64 #56 (NC, SHAI) | *If serious illness, would ruin aspects of life* | **0.67** |
| HAI-64 #57 (NC) | *If serious illness, rejected by family/friends* | 0.19 |
| HAI-64 #58 (NC, SHAI) | *If serious illness, would feel that lost dignity* | *0.33* |
| HAI-64 #59 (NC) | *If serious illness, would feel ashamed* | *0.33* |
| HAI-64 #60 (NC) | *If serious illness, difficulty losing independence* | *0.36* |
| HAI-64 #61 (NC) | *If serious illness, family/friends would not cope* | *0.30* |
| HAI-64 #62 (NC) | *If serious illness, family/friends would not care* | 0.13 |
| HAI-64 #63 (NC) | *If serious illness, no one would support* | 0.18 |
| HAI-64 #64 (NC) | *If serious illness, would be unable to cope* | **0.68** |
| IAS #1 | *Do you worry about your health?* | *** 0.92** |
| IAS #2 | *Worried about serious illness in the future* | **0.89** |
| IAS #3 | *Scared by thought of serious illness* | **0.85** |
| IAS #4 | *Worried about pain being caused by illness* | **0.87** |
| IAS #5 | *If pain persists, sees a physician* | *0.33* |
| IAS #6 | *If pain lasts, convinced of illness* | **0.83** |
| IAS #7 | *Avoids habits that may be harmful* | -0.14 |
| IAS #8 | *Avoids foods that may be unhealthy* | -0.14 |
| IAS #9 | *Examines body for disease* | **0.61** |
| IAS #10 | *Belief in physical disease not diagnosed* | **0.78** |
| IAS #11 | *Refuses to believe doctor if told no disease* | **0.56** |
| IAS #12 | *When informed by doctor, convinced of illness* | **0.69** |
| IAS #13 | *Afraid of news that remind of death* | **0.64** |
| IAS #14 | *Does the thought of death scare you?* | **0.77** |
| IAS #15 | *Afraid that may die soon* | **0.84** |
| IAS #16 | *Afraid that may have cancer* | **0.83** |
| IAS #17 | *Afraid that may have a heart disease* | **0.53** |
| IAS #18 | *Afraid that may have another serious illness* | **0.73** |
| IAS #19 | *Symptoms from reading or hearing about illness* | **0.77** |
| IAS #20 | *When noticing sensation, difficult to think of other* | **0.90** |
| IAS #21 | *When feeling a sensation, worries about it* | **0.90** |
| IAS #23 | *How often do you see a doctor?* | **0.56** |
| IAS #24 | *Number of doctors etc. seen in past year* | **0.60** |
| IAS #25 | *Number of treatments during the past year* | 0.25 |
| IAS #27 | *Do your bodily symptoms stop you from working?* | **0.50** |
| IAS #28 | *Bodily symptoms make it hard to concentrate* | **0.81** |
| IAS #29 | *Bodily symptoms are an obstacle to enjoyment* | **0.81** |
| WI-14 #1 | *Often worries about serious illness* | **0.89** |
| WI-14 #2 | *Bothered by many pains and aches* | *0.38* |
| WI-14 #3 | *Often aware of body* | **0.53** |
| WI-14 #4 | *Worries a lot about health* | **0.90** |
| WI-14 #5 | *Often has symptoms of serious illness* | **0.63** |
| WI-14 #6 | *Worries about diseases brought to attention* | **0.73** |
| WI-14 #7 | *Annoyed if told looking better* | 0.23 |
| WI-14 #8 | *Bothered by many different symptoms* | **0.68** |
| WI-14 #9 | *Easily forgets about self* | **0.62** |
| WI-14 #10 | *Hard to believe the doctor* | **0.58** |
| WI-14 #11 | *Other people not taking illness seriously* | **0.60** |
| WI-14 #12 | *Worries about health more than most people* | **0.88** |
| WI-14 #13 | *Thinks there is something seriously wrong* | **0.68** |
| WI-14 #14 | *Afraid of illness* | **0.83** |

*Note*. NC = Negative Consequences subscale. SHAI = items of the 18-item Short Health Anxiety Inventory. Note that “SHAI” without “NC” implies that items form part of the 14-item Short Health Anxiety Inventory.

*Table S2.* Factor loadings (pattern matrix) with one factor retained (67% variance explained), >0.90 marked *, ≥0.40 in bold text and ≥0.30 in italics, sorted according to factor loading.

| Item | Paraphrased text to facilitate interpretation | Loading |
| --- | --- | --- |
| IAS #1 | *Do you worry about your health?* | *** 0.92** |
| WI-14 #4 | *Worries a lot about health* | **0.90** |
| IAS #21 | *When feeling a sensation, worries about it* | **0.90** |
| IAS #20 | *When noticing sensation, difficult to think of other* | **0.90** |
| IAS #2 | *Worried about serious illness in the future* | **0.89** |
| HAI-64 #8 (SHAI) | *Fear of having a serious illness* | **0.89** |
| WI-14 #1 | *Often worries about serious illness* | **0.89** |
| WI-14 #12 | *Worries about health more than most people* | **0.88** |
| HAI-64 #16 (SHAI) | *Ability to take mind off thoughts about health* | **0.88** |
| HAI-64 #7 | *When bodily sensation, thinks it is a sign of illness* | **0.88** |
| HAI-64 #37 | *How often afraid of developing a serious illness* | **0.87** |
| IAS #4 | *Worried about pain being caused by illness* | **0.87** |
| HAI-64 #20 | *How often health worries interfere with life* | **0.86** |
| HAI-64 #4 | *When bodily sensation, worries about it* | **0.86** |
| IAS #3 | *Scared by thought of serious illness* | **0.85** |
| HAI-64 #43 (SHAI) | *If notices sensation, difficult think about other things* | **0.85** |
| HAI-64 #5 (SHAI) | *Ability to resist thoughts of illness* | **0.85** |
| HAI-64 #1 (SHAI) | *Time spent worrying about health* | **0.85** |
| HAI-64 #35 (SHAI) | *How often thinks that seriously ill* | **0.85** |
| IAS #15 | *Afraid that may die soon* | **0.84** |
| IAS #6 | *If pain lasts, convinced of illness* | **0.83** |
| IAS #16 | *Afraid that may have cancer* | **0.83** |
| HAI-64 #39 | *Fear when thinking about developing serious illness* | **0.83** |
| HAI-64 #9 (SHAI) | *How often mental images of being ill* | **0.83** |
| HAI-64 #45 (SHAI) | *Family and friends believe worry to be excessive* | **0.83** |
| HAI-64 #17 (SHAI) | *Not relieved if doctor says there is nothing wrong* | **0.83** |
| HAI-64 #10 | *Perceived probability: serious illness in near future* | **0.83** |
| WI-14 #14 | *Afraid of illness* | **0.83** |
| HAI-64 #40 | *When in pain, thinks that sign of illness* | **0.82** |
| HAI-64 #23 | *Impact of worry about health on thinking about other* | **0.82** |
| HAI-64 #14 | *If notices bodily sensation, how often focuses on it* | **0.82** |
| HAI-64 #6 | *How often worries about dying* | **0.82** |
| IAS #28 | *Bodily symptoms make it hard to concentrate* | **0.81** |
| IAS #29 | *Bodily symptoms are an obstacle to enjoyment* | **0.81** |
| HAI-64 #33 | *Degree of perceived vulnerability to serious illness* | **0.80** |
| HAI-64 #29 (SHAI) | *Perceived risk for developing a serious illness* | **0.79** |
| HAI-64 #36 | *Perceived probability: becoming ill in next few weeks* | **0.78** |
| IAS #10 | *Belief in physical disease not diagnosed* | **0.78** |
| HAI-64 #27 (SHAI) | *Need to understand bodily sensations/changes* | **0.77** |
| HAI-64 #25 | *How sensible is the idea of having serious illness* | **0.77** |
| IAS #19 | *Symptoms from reading or hearing about illness* | **0.77** |
| IAS #14 | *Does the thought of death scare you?* | **0.77** |
| HAI-64 #11 | *If notices bodily sensation, how often checks on it* | **0.76** |
| HAI-64 #21 | *How often beliefs going to die soon* | **0.76** |
| HAI-64 #47 | *Own belief in worry being excessive* | **0.76** |
| HAI-64 #44 | *If hears about illness, worries about other illness* | **0.76** |
| HAI-64 #2 (SHAI) | *Awareness of aches and pains* | **0.74** |
| HAI-64 #32 | *How often thinks about being seriously ill* | **0.74** |
| HAI-64 #3 (SHAI) | *Awareness of bodily sensations or changes* | **0.74** |
| HAI-64 #46 | *GP’s belief in worry being excessive* | **0.73** |
| IAS #18 | *Afraid that may have another serious illness* | **0.73** |
| WI-14 #6 | *Worries about diseases brought to attention* | **0.73** |
| HAI-64 #52 (NC, SHAI) | *If serious illness, unable to enjoy life* | **0.72** |
| HAI-64 #30 | *How often images of dying or being dead* | **0.71** |
| HAI-64 #48 (NC) | *If serious illness, would reduce quality of life* | **0.71** |
| HAI-64 #38 | *Perceived probability: undiagnosed serious disease* | **0.70** |
| HAI-64 #18 (SHAI) | *If hears about illness, belief in having it* | **0.69** |
| IAS #12 | *When informed by doctor, convinced of illness* | **0.69** |
| HAI-64 #24 | *If notices sensation, how often attempt at distraction* | **0.69** |
| HAI-64 #64 (NC) | *If serious illness, would be unable to cope* | **0.68** |
| WI-14 #13 | *Thinks there is something seriously wrong* | **0.68** |
| WI-14 #8 | *Bothered by many different symptoms* | **0.68** |
| HAI-64 #56 (NC, SHAI) | *If serious illness, would ruin aspects of life* | **0.67** |
| HAI-64 #53 (NC, SHAI) | *If serious illness, little belief in modern medicine* | **0.67** |
| HAI-64 #50 (NC) | *If serious illness, would be fatal* | **0.67** |
| HAI-64 #41 | *How often feels as though going to die soon* | **0.65** |
| HAI-64 #12 | *How realistic that the respondent is seriously ill* | **0.65** |
| IAS #13 | *Afraid of news that remind of death* | **0.64** |
| HAI-64 #51 (NC) | *If serious illness, prolonged suffering* | **0.64** |
| WI-14 #5 | *Often has symptoms of serious illness* | **0.63** |
| HAI-64 #19 | *If notices sensations, tries to find the cause* | **0.63** |
| WI-14 #9 | *Easily forgets about self* | **0.62** |
| HAI-64 #13 | *Avoidance of situations that trigger thoughts of death* | **0.62** |
| HAI-64 #15 | *How often examines body* | **0.62** |
| IAS #9 | *Examines body for disease* | **0.61** |
| HAI-64 #22 | *Afraid of visiting doctor because of health worries* | **0.60** |
| WI-14 #11 | *Other people not taking illness seriously* | **0.60** |
| IAS #24 | *Number of doctors etc. seen in past year* | **0.60** |
| WI-14 #10 | *Hard to believe the doctor* | **0.58** |
| HAI-64 #28 | *Avoids situations where illness is prominent* | **0.58** |
| IAS #23 | *How often do you see a doctor?* | **0.56** |
| IAS #11 | *Refuses to believe doctor if told no disease* | **0.56** |
| HAI-64 #49 (NC) | *If serious illness, be very painful* | **0.56** |
| IAS #17 | *Afraid that may have a heart disease* | **0.53** |
| WI-14 #3 | *Often aware of body* | **0.53** |
| IAS #27 | *Do your bodily symptoms stop you from working?* | **0.50** |
| HAI-64 #34 | *If notices sensation, how often attempts reassurance* | **0.50** |
| HAI-64 #42 | *If notices sensation, tries to get rid of it* | **0.44** |
| HAI-64 #31 | *How often mentions unexplained bodily sensations* | **0.41** |
| WI-14 #2 | *Bothered by many pains and aches* | *0.38* |
| HAI-64 #60 (NC) | *If serious illness, difficulty losing independence* | *0.36* |
| HAI-64 #55 (NC) | *If serious illness, reduced belief in own worth* | *0.36* |
| HAI-64 #26 | *Previous illnesses mismanaged* | *0.33* |
| HAI-64 #59 (NC) | *If serious illness, would feel ashamed* | *0.33* |
| IAS #5 | *If pain persists, sees a physician* | *0.33* |
| HAI-64 #58 (NC, SHAI) | *If serious illness, would feel that lost dignity* | *0.33* |
| HAI-64 #61 (NC) | *If serious illness, family/friends would not cope* | *0.30* |
| IAS #25 | *Number of treatments during the past year* | 0.25 |
| WI-14 #7 | *Annoyed if told looking better* | 0.23 |
| HAI-64 #54 (NC) | *If serious illness, family/friends would express pity* | 0.23 |
| HAI-64 #57 (NC) | *If serious illness, rejected by family/friends* | 0.19 |
| HAI-64 #63 (NC) | *If serious illness, no one would support* | 0.18 |
| HAI-64 #62 (NC) | *If serious illness, family/friends would not care* | 0.13 |
| IAS #7 | *Avoids habits that may be harmful* | -0.14 |
| IAS #8 | *Avoids foods that may be unhealthy* | -0.14 |

*Note*. NC = Negative Consequences subscale. SHAI = items of the 18-item Short Health Anxiety Inventory. Note that “SHAI” without “NC” implies that items form part of the 14-item Short Health Anxiety Inventory.

*Table S3.* Factor loadings (pattern matrix) with two factors retained (67% vs. 25% variance explained), >0.90 marked *, ≥0.40 in bold text and ≥0.30 in italics, after promax rotation, sorted according to scale.

| Item | Paraphrased text to facilitate interpretation | Factor 1 | Factor 2 |
| --- | --- | --- | --- |
| HAI-64 #1 (SHAI) | *Time spent worrying about health* | **0.83** | 0.04 |
| HAI-64 #2 (SHAI) | *Awareness of aches and pains* | **0.75** | -0.02 |
| HAI-64 #3 (SHAI) | *Awareness of bodily sensations or changes* | **0.75** | -0.02 |
| HAI-64 #4 | *When bodily sensation, worries about it* | *** 0.91** | -0.10 |
| HAI-64 #5 (SHAI) | *Ability to resist thoughts of illness* | **0.88** | -0.05 |
| HAI-64 #6 | *How often worries about dying* | **0.85** | -0.06 |
| HAI-64 #7 | *When bodily sensation, thinks it is a sign of illness* | *** 0.92** | -0.07 |
| HAI-64 #8 (SHAI) | *Fear of having a serious illness* | *** 0.94** | -0.09 |
| HAI-64 #9 (SHAI) | *How often mental images of being ill* | **0.80** | 0.06 |
| HAI-64 #10 | *Perceived probability: serious illness in near future* | **0.83** | 0.00 |
| HAI-64 #11 | *If notices bodily sensation, how often checks on it* | **0.79** | -0.04 |
| HAI-64 #12 | *How realistic that the respondent is seriously ill* | **0.61** | 0.08 |
| HAI-64 #13 | *Avoidance of situations that trigger thoughts of death* | **0.55** | 0.13 |
| HAI-64 #14 | *If notices bodily sensation, how often focuses on it* | **0.83** | -0.02 |
| HAI-64 #15 | *How often examines body* | **0.58** | 0.06 |
| HAI-64 #16 (SHAI) | *Ability to take mind off thoughts about health* | **0.85** | 0.06 |
| HAI-64 #17 (SHAI) | *Not relieved if doctor says there is nothing wrong* | **0.83** | 0.00 |
| HAI-64 #18 (SHAI) | *If hears about illness, belief in having it* | **0.63** | 0.12 |
| HAI-64 #19 | *If notices sensations, tries to find the cause* | **0.67** | -0.07 |
| HAI-64 #20 | *How often health worries interfere with life* | **0.84** | 0.04 |
| HAI-64 #21 | *How often beliefs going to die soon* | **0.80** | -0.07 |
| HAI-64 #22 | *Afraid of visiting doctor because of health worries* | **0.49** | 0.19 |
| HAI-64 #23 | *Impact of worry about health on thinking about other* | **0.80** | 0.03 |
| HAI-64 #24 | *If notices sensation, how often attempt at distraction* | **0.69** | -0.01 |
| HAI-64 #25 | *How sensible is the idea of having serious illness* | **0.74** | 0.06 |
| HAI-64 #26 | *Previous illnesses mismanaged* | 0.26 | 0.14 |
| HAI-64 #27 (SHAI) | *Need to understand bodily sensations/changes* | **0.76** | 0.03 |
| HAI-64 #28 | *Avoids situations where illness is prominent* | **0.45** | 0.22 |
| HAI-64 #29 (SHAI) | *Perceived risk for developing a serious illness* | **0.77** | 0.04 |
| HAI-64 #30 | *How often images of dying or being dead* | **0.69** | 0.03 |
| HAI-64 #31 | *How often mentions unexplained bodily sensations* | **0.47** | -0.10 |
| HAI-64 #32 | *How often thinks about being seriously ill* | **0.70** | 0.07 |
| HAI-64 #33 | *Degree of perceived vulnerability to serious illness* | **0.78** | 0.04 |
| HAI-64 #34 | *If notices sensation, how often attempts reassurance* | **0.56** | -0.10 |
| HAI-64 #35 (SHAI) | *How often thinks that seriously ill* | **0.85** | -0.01 |
| HAI-64 #36 | *Perceived probability: becoming ill in next few weeks* | **0.72** | 0.11 |
| HAI-64 #37 | *How often afraid of developing a serious illness* | **0.86** | 0.02 |
| HAI-64 #38 | *Perceived probability: undiagnosed serious disease* | **0.62** | 0.13 |
| HAI-64 #39 | *Fear when thinking about developing serious illness* | **0.80** | 0.06 |
| HAI-64 #40 | *When in pain, thinks that sign of illness* | **0.84** | -0.03 |
| HAI-64 #41 | *How often feels as though going to die soon* | **0.63** | 0.05 |
| HAI-64 #42 | *If notices sensation, tries to get rid of it* | **0.40** | 0.08 |
| HAI-64 #43 (SHAI) | *If notices sensation, difficult think about other things* | **0.84** | 0.03 |
| HAI-64 #44 | *If hears about illness, worries about other illness* | **0.70** | 0.10 |
| HAI-64 #45 (SHAI) | *Family and friends believe worry to be excessive* | **0.84** | -0.01 |
| HAI-64 #46 | *GP’s belief in worry being excessive* | **0.76** | -0.05 |
| HAI-64 #47 | *Own belief in worry being excessive* | **0.81** | -0.09 |
| HAI-64 #48 (NC) | *If serious illness, would reduce quality of life* | **0.49** | *0.38* |
| HAI-64 #49 (NC) | *If serious illness, be very painful* | *0.35* | *0.37* |
| HAI-64 #50 (NC) | *If serious illness, would be fatal* | **0.58** | 0.16 |
| HAI-64 #51 (NC) | *If serious illness, prolonged suffering* | **0.42** | *0.37* |
| HAI-64 #52 (NC, SHAI) | *If serious illness, unable to enjoy life* | **0.53** | *0.33* |
| HAI-64 #53 (NC, SHAI) | *If serious illness, little belief in modern medicine* | **0.53** | 0.26 |
| HAI-64 #54 (NC) | *If serious illness, family/friends would express pity* | -0.17 | **0.69** |
| HAI-64 #55 (NC) | *If serious illness, reduced belief in own worth* | -0.05 | **0.72** |
| HAI-64 #56 (NC, SHAI) | *If serious illness, would ruin aspects of life* | **0.45** | **0.40** |
| HAI-64 #57 (NC) | *If serious illness, rejected by family/friends* | -0.21 | **0.69** |
| HAI-64 #58 (NC, SHAI) | *If serious illness, would feel that lost dignity* | -0.09 | **0.74** |
| HAI-64 #59 (NC) | *If serious illness, would feel ashamed* | -0.04 | **0.66** |
| HAI-64 #60 (NC) | *If serious illness, difficulty losing independence* | 0.12 | **0.43** |
| HAI-64 #61 (NC) | *If serious illness, family/friends would not cope* | 0.29 | 0.02 |
| HAI-64 #62 (NC) | *If serious illness, family/friends would not care* | -0.20 | **0.59** |
| HAI-64 #63 (NC) | *If serious illness, no one would support* | -0.12 | **0.53** |
| HAI-64 #64 (NC) | *If serious illness, would be unable to cope* | **0.49** | *0.35* |
| IAS #1 | *Do you worry about your health?* | *** 0.95** | -0.05 |
| IAS #2 | *Worried about serious illness in the future* | **0.89** | 0.01 |
| IAS #3 | *Scared by thought of serious illness* | **0.87** | -0.03 |
| IAS #4 | *Worried about pain being caused by illness* | *** 0.92** | -0.09 |
| IAS #5 | *If pain persists, sees a physician* | *0.39* | -0.10 |
| IAS #6 | *If pain lasts, convinced of illness* | *** 0.92** | -0.14 |
| IAS #7 | *Avoids habits that may be harmful* | -0.20 | 0.12 |
| IAS #8 | *Avoids foods that may be unhealthy* | -0.26 | 0.20 |
| IAS #9 | *Examines body for disease* | **0.59** | 0.04 |
| IAS #10 | *Belief in physical disease not diagnosed* | **0.80** | -0.03 |
| IAS #11 | *Refuses to believe doctor if told no disease* | **0.50** | 0.10 |
| IAS #12 | *When informed by doctor, convinced of illness* | **0.62** | 0.14 |
| IAS #13 | *Afraid of news that remind of death* | **0.59** | 0.10 |
| IAS #14 | *Does the thought of death scare you?* | **0.78** | -0.02 |
| IAS #15 | *Afraid that may die soon* | **0.88** | -0.08 |
| IAS #16 | *Afraid that may have cancer* | **0.87** | -0.06 |
| IAS #17 | *Afraid that may have a heart disease* | **0.51** | 0.04 |
| IAS #18 | *Afraid that may have another serious illness* | **0.67** | 0.11 |
| IAS #19 | *Symptoms from reading or hearing about illness* | **0.71** | 0.12 |
| IAS #20 | *When noticing sensation, difficult to think of other* | *** 0.94** | -0.07 |
| IAS #21 | *When feeling a sensation, worries about it* | *** 0.93** | -0.06 |
| IAS #23 | *How often do you see a doctor?* | **0.62** | -0.10 |
| IAS #24 | *Number of doctors etc. seen in past year* | **0.62** | -0.04 |
| IAS #25 | *Number of treatments during the past year* | 0.28 | -0.04 |
| IAS #27 | *Do your bodily symptoms stop you from working?* | **0.41** | 0.16 |
| IAS #28 | *Bodily symptoms make it hard to concentrate* | **0.75** | 0.10 |
| IAS #29 | *Bodily symptoms are an obstacle to enjoyment* | **0.72** | 0.15 |
| WI-14 #1 | *Often worries about serious illness* | *** 0.94** | -0.08 |
| WI-14 #2 | *Bothered by many pains and aches* | *0.38* | 0.00 |
| WI-14 #3 | *Often aware of body* | **0.58** | -0.09 |
| WI-14 #4 | *Worries a lot about health* | *** 0.95** | -0.08 |
| WI-14 #5 | *Often has symptoms of serious illness* | **0.62** | 0.01 |
| WI-14 #6 | *Worries about diseases brought to attention* | **0.70** | 0.04 |
| WI-14 #7 | *Annoyed if told looking better* | 0.14 | 0.16 |
| WI-14 #8 | *Bothered by many different symptoms* | **0.71** | -0.05 |
| WI-14 #9 | *Easily forgets about self* | **0.68** | -0.10 |
| WI-14 #10 | *Hard to believe the doctor* | **0.54** | 0.08 |
| WI-14 #11 | *Other people not taking illness seriously* | **0.54** | 0.11 |
| WI-14 #12 | *Worries about health more than most people* | *** 0.94** | -0.10 |
| WI-14 #13 | *Thinks there is something seriously wrong* | **0.66** | 0.05 |
| WI-14 #14 | *Afraid of illness* | **0.84** | -0.02 |

*Note*. NC = Negative Consequences subscale. SHAI = items of the 18-item Short Health Anxiety Inventory. Note that “SHAI” without “NC” implies that items form part of the 14-item Short Health Anxiety Inventory.

*Table S4.* Factor loadings (pattern matrix) with two factors retained (67% vs. 25% variance explained), >0.90 marked *, ≥0.40 in bold text and ≥0.30 in italics, after promax rotation, sorted according to factor loading.

| Item | Paraphrased text to facilitate interpretation | Factor 1 | Factor 2 |
| --- | --- | --- | --- |
| IAS #1 | *Do you worry about your health?* | *** 0.95** | -0.05 |
| WI-14 #4 | *Worries a lot about health* | *** 0.95** | -0.08 |
| IAS #20 | *When noticing sensation, difficult to think of other* | *** 0.94** | -0.07 |
| WI-14 #1 | *Often worries about serious illness* | *** 0.94** | -0.08 |
| HAI-64 #8 (SHAI) | *Fear of having a serious illness* | *** 0.94** | -0.09 |
| WI-14 #12 | *Worries about health more than most people* | *** 0.94** | -0.10 |
| IAS #21 | *When feeling a sensation, worries about it* | *** 0.93** | -0.06 |
| HAI-64 #7 | *When bodily sensation, thinks it is a sign of illness* | *** 0.92** | -0.07 |
| IAS #4 | *Worried about pain being caused by illness* | *** 0.92** | -0.09 |
| IAS #6 | *If pain lasts, convinced of illness* | *** 0.92** | -0.14 |
| HAI-64 #4 | *When bodily sensation, worries about it* | *** 0.91** | -0.10 |
| IAS #2 | *Worried about serious illness in the future* | **0.89** | 0.01 |
| IAS #15 | *Afraid that may die soon* | **0.88** | -0.08 |
| HAI-64 #5 (SHAI) | *Ability to resist thoughts of illness* | **0.88** | -0.05 |
| IAS #16 | *Afraid that may have cancer* | **0.87** | -0.06 |
| IAS #3 | *Scared by thought of serious illness* | **0.87** | -0.03 |
| HAI-64 #37 | *How often afraid of developing a serious illness* | **0.86** | 0.02 |
| HAI-64 #35 (SHAI) | *How often thinks that seriously ill* | **0.85** | -0.01 |
| HAI-64 #6 | *How often worries about dying* | **0.85** | -0.06 |
| HAI-64 #16 (SHAI) | *Ability to take mind off thoughts about health* | **0.85** | 0.06 |
| HAI-64 #40 | *When in pain, thinks that sign of illness* | **0.84** | -0.03 |
| HAI-64 #45 (SHAI) | *Family and friends believe worry to be excessive* | **0.84** | -0.01 |
| WI-14 #14 | *Afraid of illness* | **0.84** | -0.02 |
| HAI-64 #20 | *How often health worries interfere with life* | **0.84** | 0.04 |
| HAI-64 #43 (SHAI) | *If notices sensation, difficult think about other things* | **0.84** | 0.03 |
| HAI-64 #17 (SHAI) | *Not relieved if doctor says there is nothing wrong* | **0.83** | 0.00 |
| HAI-64 #10 | *Perceived probability: serious illness in near future* | **0.83** | 0.00 |
| HAI-64 #14 | *If notices bodily sensation, how often focuses on it* | **0.83** | -0.02 |
| HAI-64 #1 (SHAI) | *Time spent worrying about health* | **0.83** | 0.04 |
| HAI-64 #47 | *Own belief in worry being excessive* | **0.81** | -0.09 |
| HAI-64 #21 | *How often beliefs going to die soon* | **0.80** | -0.07 |
| IAS #10 | *Belief in physical disease not diagnosed* | **0.80** | -0.03 |
| HAI-64 #39 | *Fear when thinking about developing serious illness* | **0.80** | 0.06 |
| HAI-64 #23 | *Impact of worry about health on thinking about other* | **0.80** | 0.03 |
| HAI-64 #9 (SHAI) | *How often mental images of being ill* | **0.80** | 0.06 |
| HAI-64 #11 | *If notices bodily sensation, how often checks on it* | **0.79** | -0.04 |
| HAI-64 #33 | *Degree of perceived vulnerability to serious illness* | **0.78** | 0.04 |
| IAS #14 | *Does the thought of death scare you?* | **0.78** | -0.02 |
| HAI-64 #29 (SHAI) | *Perceived risk for developing a serious illness* | **0.77** | 0.04 |
| HAI-64 #46 | *GP’s belief in worry being excessive* | **0.76** | -0.05 |
| HAI-64 #27 (SHAI) | *Need to understand bodily sensations/changes* | **0.76** | 0.03 |
| HAI-64 #2 (SHAI) | *Awareness of aches and pains* | **0.75** | -0.02 |
| IAS #28 | *Bodily symptoms make it hard to concentrate* | **0.75** | 0.10 |
| HAI-64 #3 (SHAI) | *Awareness of bodily sensations or changes* | **0.75** | -0.02 |
| HAI-64 #25 | *How sensible is the idea of having serious illness* | **0.74** | 0.06 |
| HAI-64 #36 | *Perceived probability: becoming ill in next few weeks* | **0.72** | 0.11 |
| IAS #29 | *Bodily symptoms are an obstacle to enjoyment* | **0.72** | 0.15 |
| IAS #19 | *Symptoms from reading or hearing about illness* | **0.71** | 0.12 |
| WI-14 #8 | *Bothered by many different symptoms* | **0.71** | -0.05 |
| WI-14 #6 | *Worries about diseases brought to attention* | **0.70** | 0.04 |
| HAI-64 #32 | *How often thinks about being seriously ill* | **0.70** | 0.07 |
| HAI-64 #44 | *If hears about illness, worries about other illness* | **0.70** | 0.10 |
| HAI-64 #30 | *How often images of dying or being dead* | **0.69** | 0.03 |
| HAI-64 #24 | *If notices sensation, how often attempt at distraction* | **0.69** | -0.01 |
| WI-14 #9 | *Easily forgets about self* | **0.68** | -0.10 |
| IAS #18 | *Afraid that may have another serious illness* | **0.67** | 0.11 |
| HAI-64 #19 | *If notices sensations, tries to find the cause* | **0.67** | -0.07 |
| WI-14 #13 | *Thinks there is something seriously wrong* | **0.66** | 0.05 |
| HAI-64 #18 (SHAI) | *If hears about illness, belief in having it* | **0.63** | 0.12 |
| HAI-64 #41 | *How often feels as though going to die soon* | **0.63** | 0.05 |
| WI-14 #5 | *Often has symptoms of serious illness* | **0.62** | 0.01 |
| HAI-64 #38 | *Perceived probability: undiagnosed serious disease* | **0.62** | 0.13 |
| IAS #24 | *Number of doctors etc. seen in past year* | **0.62** | -0.04 |
| IAS #23 | *How often do you see a doctor?* | **0.62** | -0.10 |
| IAS #12 | *When informed by doctor, convinced of illness* | **0.62** | 0.14 |
| HAI-64 #12 | *How realistic that the respondent is seriously ill* | **0.61** | 0.08 |
| IAS #13 | *Afraid of news that remind of death* | **0.59** | 0.10 |
| IAS #9 | *Examines body for disease* | **0.59** | 0.04 |
| HAI-64 #15 | *How often examines body* | **0.58** | 0.06 |
| WI-14 #3 | *Often aware of body* | **0.58** | -0.09 |
| HAI-64 #50 (NC) | *If serious illness, would be fatal* | **0.58** | 0.16 |
| HAI-64 #34 | *If notices sensation, how often attempts reassurance* | **0.56** | -0.10 |
| HAI-64 #13 | *Avoidance of situations that trigger thoughts of death* | **0.55** | 0.13 |
| WI-14 #11 | *Other people not taking illness seriously* | **0.54** | 0.11 |
| WI-14 #10 | *Hard to believe the doctor* | **0.54** | 0.08 |
| HAI-64 #52 (NC, SHAI) | *If serious illness, unable to enjoy life* | **0.53** | *0.33* |
| HAI-64 #53 (NC, SHAI) | *If serious illness, little belief in modern medicine* | **0.53** | 0.26 |
| IAS #17 | *Afraid that may have a heart disease* | **0.51** | 0.04 |
| IAS #11 | *Refuses to believe doctor if told no disease* | **0.50** | 0.10 |
| HAI-64 #22 | *Afraid of visiting doctor because of health worries* | **0.49** | 0.19 |
| HAI-64 #48 (NC) | *If serious illness, would reduce quality of life* | **0.49** | *0.38* |
| HAI-64 #64 (NC) | *If serious illness, would be unable to cope* | **0.49** | *0.35* |
| HAI-64 #31 | *How often mentions unexplained bodily sensations* | **0.47** | -0.10 |
| HAI-64 #28 | *Avoids situations where illness is prominent* | **0.45** | 0.22 |
| HAI-64 #56 (NC, SHAI) | *If serious illness, would ruin aspects of life* | **0.45** | **0.40** |
| HAI-64 #51 (NC) | *If serious illness, prolonged suffering* | **0.42** | *0.37* |
| IAS #27 | *Do your bodily symptoms stop you from working?* | **0.41** | 0.16 |
| HAI-64 #42 | *If notices sensation, tries to get rid of it* | **0.40** | 0.08 |
| IAS #5 | *If pain persists, sees a physician* | *0.39* | -0.10 |
| WI-14 #2 | *Bothered by many pains and aches* | *0.38* | 0.00 |
| HAI-64 #49 (NC) | *If serious illness, be very painful* | *0.35* | *0.37* |
| HAI-64 #61 (NC) | *If serious illness, family/friends would not cope* | 0.29 | 0.02 |
| IAS #25 | *Number of treatments during the past year* | 0.28 | -0.04 |
| HAI-64 #26 | *Previous illnesses mismanaged* | 0.26 | 0.14 |
| WI-14 #7 | *Annoyed if told looking better* | 0.14 | 0.16 |
| HAI-64 #60 (NC) | *If serious illness, difficulty losing independence* | 0.12 | **0.43** |
| HAI-64 #59 (NC) | *If serious illness, would feel ashamed* | -0.04 | **0.66** |
| HAI-64 #55 (NC) | *If serious illness, reduced belief in own worth* | -0.05 | **0.72** |
| HAI-64 #58 (NC, SHAI) | *If serious illness, would feel that lost dignity* | -0.09 | **0.74** |
| HAI-64 #63 (NC) | *If serious illness, no one would support* | -0.12 | **0.53** |
| HAI-64 #54 (NC) | *If serious illness, family/friends would express pity* | -0.17 | **0.69** |
| HAI-64 #62 (NC) | *If serious illness, family/friends would not care* | -0.20 | **0.59** |
| IAS #7 | *Avoids habits that may be harmful* | -0.20 | 0.12 |
| HAI-64 #57 (NC) | *If serious illness, rejected by family/friends* | -0.21 | **0.69** |
| IAS #8 | *Avoids foods that may be unhealthy* | -0.26 | 0.20 |

*Note*. NC = Negative Consequences subscale. SHAI = items of the 18-item Short Health Anxiety Inventory. Note that “SHAI” without “NC” implies that items form part of the 14-item Short Health Anxiety Inventory.

### *Part II: Results from joint factor analysis of the 14-item Health Anxiety Inventory (HAI-14), the Illness Attitude Scale (IAS), and the 14-item Whiteley Index with yes/no items (WI-14)*


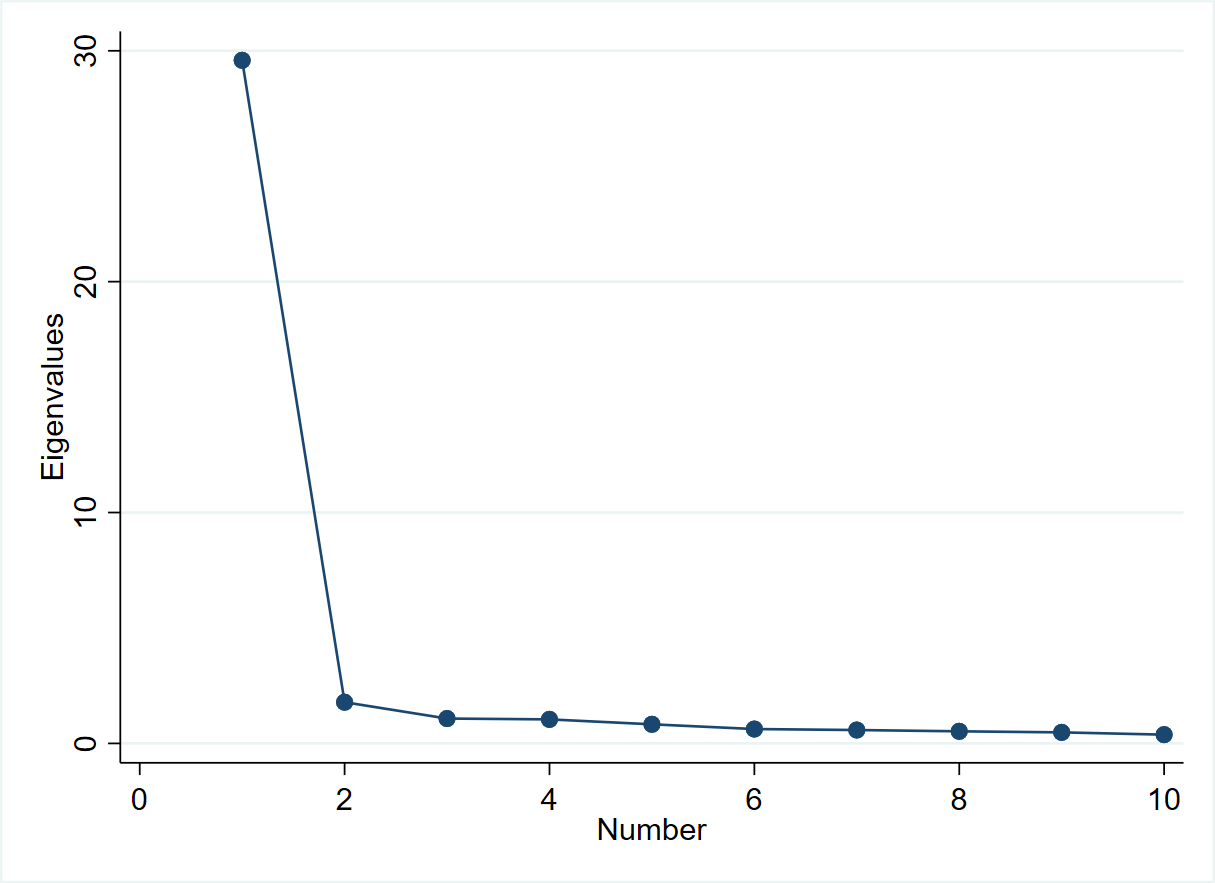


*Figure S2.* Sensitivity analysis using only the HAI-14, IAS, and WI-14. Scree plot of 10 highest eigenvalues.

*Table S5.* Sensitivity analysis using only the HAI-14, IAS, and WI-14. Factor loadings (pattern matrix) with one factor retained (79% variance explained), >0.90 marked *, ≥0.40 in bold text and ≥0.30 in italics, sorted according to scale.

| Item | Paraphrased text to facilitate interpretation | Loading |
| --- | --- | --- |
| HAI-64 #1 (SHAI) | *Time spent worrying about health* | **0.85** |
| HAI-64 #2 (SHAI) | *Awareness of aches and pains* | **0.75** |
| HAI-64 #3 (SHAI) | *Awareness of bodily sensations or changes* | **0.74** |
| HAI-64 #5 (SHAI) | *Ability to resist thoughts of illness* | **0.86** |
| HAI-64 #8 (SHAI) | *Fear of having a serious illness* | **0.89** |
| HAI-64 #9 (SHAI) | *How often mental images of being ill* | **0.82** |
| HAI-64 #16 (SHAI) | *Ability to take mind off thoughts about health* | **0.88** |
| HAI-64 #17 (SHAI) | *Not relieved if doctor says there is nothing wrong* | **0.84** |
| HAI-64 #18 (SHAI) | *If hears about illness, belief in having it* | **0.68** |
| HAI-64 #27 (SHAI) | *Need to understand bodily sensations/changes* | **0.76** |
| HAI-64 #29 (SHAI) | *Perceived risk for developing a serious illness* | **0.77** |
| HAI-64 #35 (SHAI) | *How often thinks that seriously ill* | **0.84** |
| HAI-64 #43 (SHAI) | *If notices sensation, difficult think about other things* | **0.84** |
| HAI-64 #45 (SHAI) | *Family and friends believe worry to be excessive* | **0.83** |
| IAS #1 | *Do you worry about your health?* | *** 0.93** |
| IAS #2 | *Worried about serious illness in the future* | **0.89** |
| IAS #3 | *Scared by thought of serious illness* | **0.86** |
| IAS #4 | *Worried about pain being caused by illness* | **0.88** |
| IAS #5 | *If pain persists, sees a physician* | *0.34* |
| IAS #6 | *If pain lasts, convinced of illness* | **0.84** |
| IAS #7 | *Avoids habits that may be harmful* | -0.15 |
| IAS #8 | *Avoids foods that may be unhealthy* | -0.15 |
| IAS #9 | *Examines body for disease* | **0.60** |
| IAS #10 | *Belief in physical disease not diagnosed* | **0.79** |
| IAS #11 | *Refuses to believe doctor if told no disease* | **0.57** |
| IAS #12 | *When informed by doctor, convinced of illness* | **0.69** |
| IAS #13 | *Afraid of news that remind of death* | **0.63** |
| IAS #14 | *Does the thought of death scare you?* | **0.76** |
| IAS #15 | *Afraid that may die soon* | **0.83** |
| IAS #16 | *Afraid that may have cancer* | **0.83** |
| IAS #17 | *Afraid that may have a heart disease* | **0.55** |
| IAS #18 | *Afraid that may have another serious illness* | **0.73** |
| IAS #19 | *Symptoms from reading or hearing about illness* | **0.78** |
| IAS #20 | *When noticing sensation, difficult to think of other* | *** 0.91** |
| IAS #21 | *When feeling a sensation, worries about it* | *** 0.91** |
| IAS #23 | *How often do you see a doctor?* | **0.58** |
| IAS #24 | *Number of doctors etc. seen in past year* | **0.61** |
| IAS #25 | *Number of treatments during the past year* | 0.26 |
| IAS #27 | *Do your bodily symptoms stop you from working?* | **0.51** |
| IAS #28 | *Bodily symptoms make it hard to concentrate* | **0.82** |
| IAS #29 | *Bodily symptoms are an obstacle to enjoyment* | **0.82** |
| WI-14 #1 | *Often worries about serious illness* | *** 0.91** |
| WI-14 #2 | *Bothered by many pains and aches* | **0.40** |
| WI-14 #3 | *Often aware of body* | **0.54** |
| WI-14 #4 | *Worries a lot about health* | *** 0.92** |
| WI-14 #5 | *Often has symptoms of serious illness* | **0.64** |
| WI-14 #6 | *Worries about diseases brought to attention* | **0.73** |
| WI-14 #7 | *Annoyed if told looking better* | 0.23 |
| WI-14 #8 | *Bothered by many different symptoms* | **0.70** |
| WI-14 #9 | *Easily forgets about self* | **0.63** |
| WI-14 #10 | *Hard to believe the doctor* | **0.59** |
| WI-14 #11 | *Other people not taking illness seriously* | **0.61** |
| WI-14 #12 | *Worries about health more than most people* | **0.90** |
| WI-14 #13 | *Thinks there is something seriously wrong* | **0.69** |
| WI-14 #14 | *Afraid of illness* | **0.84** |

*Note*. NC = Negative Consequences subscale. SHAI = items of the 18-item Short Health Anxiety Inventory. Note that “SHAI” without “NC” implies that items form part of the 14-item Short Health Anxiety Inventory.

*Table S6.* Sensitivity analysis using only the HAI-14, IAS, and WI-14. Factor loadings (pattern matrix) with one factor retained 79% variance explained), >0.90 marked *, ≥0.40 in bold text and ≥0.30 in italics, sorted according to factor loading.

| Item | Paraphrased text to facilitate interpretation | Loading |
| --- | --- | --- |
| IAS #1 | *Do you worry about your health?* | *** 0.93** |
| WI-14 #4 | *Worries a lot about health* | *** 0.92** |
| IAS #20 | *When noticing sensation, difficult to think of other* | *** 0.91** |
| IAS #21 | *When feeling a sensation, worries about it* | *** 0.91** |
| WI-14 #1 | *Often worries about serious illness* | *** 0.91** |
| WI-14 #12 | *Worries about health more than most people* | **0.90** |
| IAS #2 | *Worried about serious illness in the future* | **0.89** |
| HAI-64 #8 (SHAI) | *Fear of having a serious illness* | **0.89** |
| IAS #4 | *Worried about pain being caused by illness* | **0.88** |
| HAI-64 #16 (SHAI) | *Ability to take mind off thoughts about health* | **0.88** |
| HAI-64 #5 (SHAI) | *Ability to resist thoughts of illness* | **0.86** |
| IAS #3 | *Scared by thought of serious illness* | **0.86** |
| HAI-64 #1 (SHAI) | *Time spent worrying about health* | **0.85** |
| HAI-64 #43 (SHAI) | *If notices sensation, difficult think about other things* | **0.84** |
| IAS #6 | *If pain lasts, convinced of illness* | **0.84** |
| HAI-64 #17 (SHAI) | *Not relieved if doctor says there is nothing wrong* | **0.84** |
| WI-14 #14 | *Afraid of illness* | **0.84** |
| HAI-64 #35 (SHAI) | *How often thinks that seriously ill* | **0.84** |
| IAS #16 | *Afraid that may have cancer* | **0.83** |
| IAS #15 | *Afraid that may die soon* | **0.83** |
| HAI-64 #45 (SHAI) | *Family and friends believe worry to be excessive* | **0.83** |
| IAS #28 | *Bodily symptoms make it hard to concentrate* | **0.82** |
| HAI-64 #9 (SHAI) | *How often mental images of being ill* | **0.82** |
| IAS #29 | *Bodily symptoms are an obstacle to enjoyment* | **0.82** |
| IAS #10 | *Belief in physical disease not diagnosed* | **0.79** |
| IAS #19 | *Symptoms from reading or hearing about illness* | **0.78** |
| HAI-64 #29 (SHAI) | *Perceived risk for developing a serious illness* | **0.77** |
| IAS #14 | *Does the thought of death scare you?* | **0.76** |
| HAI-64 #27 (SHAI) | *Need to understand bodily sensations/changes* | **0.76** |
| HAI-64 #2 (SHAI) | *Awareness of aches and pains* | **0.75** |
| HAI-64 #3 (SHAI) | *Awareness of bodily sensations or changes* | **0.74** |
| IAS #18 | *Afraid that may have another serious illness* | **0.73** |
| WI-14 #6 | *Worries about diseases brought to attention* | **0.73** |
| WI-14 #8 | *Bothered by many different symptoms* | **0.70** |
| IAS #12 | *When informed by doctor, convinced of illness* | **0.69** |
| WI-14 #13 | *Thinks there is something seriously wrong* | **0.69** |
| HAI-64 #18 (SHAI) | *If hears about illness, belief in having it* | **0.68** |
| WI-14 #5 | *Often has symptoms of serious illness* | **0.64** |
| WI-14 #9 | *Easily forgets about self* | **0.63** |
| IAS #13 | *Afraid of news that remind of death* | **0.63** |
| IAS #24 | *Number of doctors etc. seen in past year* | **0.61** |
| WI-14 #11 | *Other people not taking illness seriously* | **0.61** |
| IAS #9 | *Examines body for disease* | **0.60** |
| WI-14 #10 | *Hard to believe the doctor* | **0.59** |
| IAS #23 | *How often do you see a doctor?* | **0.58** |
| IAS #11 | *Refuses to believe doctor if told no disease* | **0.57** |
| IAS #17 | *Afraid that may have a heart disease* | **0.55** |
| WI-14 #3 | *Often aware of body* | **0.54** |
| IAS #27 | *Do your bodily symptoms stop you from working?* | **0.51** |
| WI-14 #2 | *Bothered by many pains and aches* | **0.40** |
| IAS #5 | *If pain persists, sees a physician* | *0.34* |
| IAS #25 | *Number of treatments during the past year* | 0.26 |
| WI-14 #7 | *Annoyed if told looking better* | 0.23 |
| IAS #8 | *Avoids foods that may be unhealthy* | -0.15 |
| IAS #7 | *Avoids habits that may be harmful* | -0.15 |

*Note*. NC = Negative Consequences subscale. SHAI = items of the 18-item Short Health Anxiety Inventory. Note that “SHAI” without “NC” implies that items form part of the 14-item Short Health Anxiety Inventory.
