# Supplementary material for: Joint factor analysis and approximate equipercentile linking of common trait health anxiety measures: a cross-sectional study of the 14-, 18- and 64-item health anxiety inventory, the illness attitude scale, and the 14-item Whiteley Index
Source: BMC Psychiatry. 2023 Sep 6;23:658. doi: 10.1186/s12888-023-05151-7 (PMC10483785; doi:10.1186/s12888-023-05151-7)
Supplement: Supplementary file 2 — Supplementary Material 2: Trait health anxiety sum score distributions [file 12888_2023_5151_MOESM2_ESM.docx]

# Supplementary material:

# Trait health anxiety sum score distributions

## Supplement of “Joint factor analysis and approximate equipercentile linking of common trait health anxiety measures: A cross-sectional study of the 14-, 18- and 64-item Health Anxiety Inventory, the Illness Attitude Scale, and the 14-item Whiteley Index”

### *Sum score distribution of the 64-item Health Anxiety Inventory (HAI-64)*


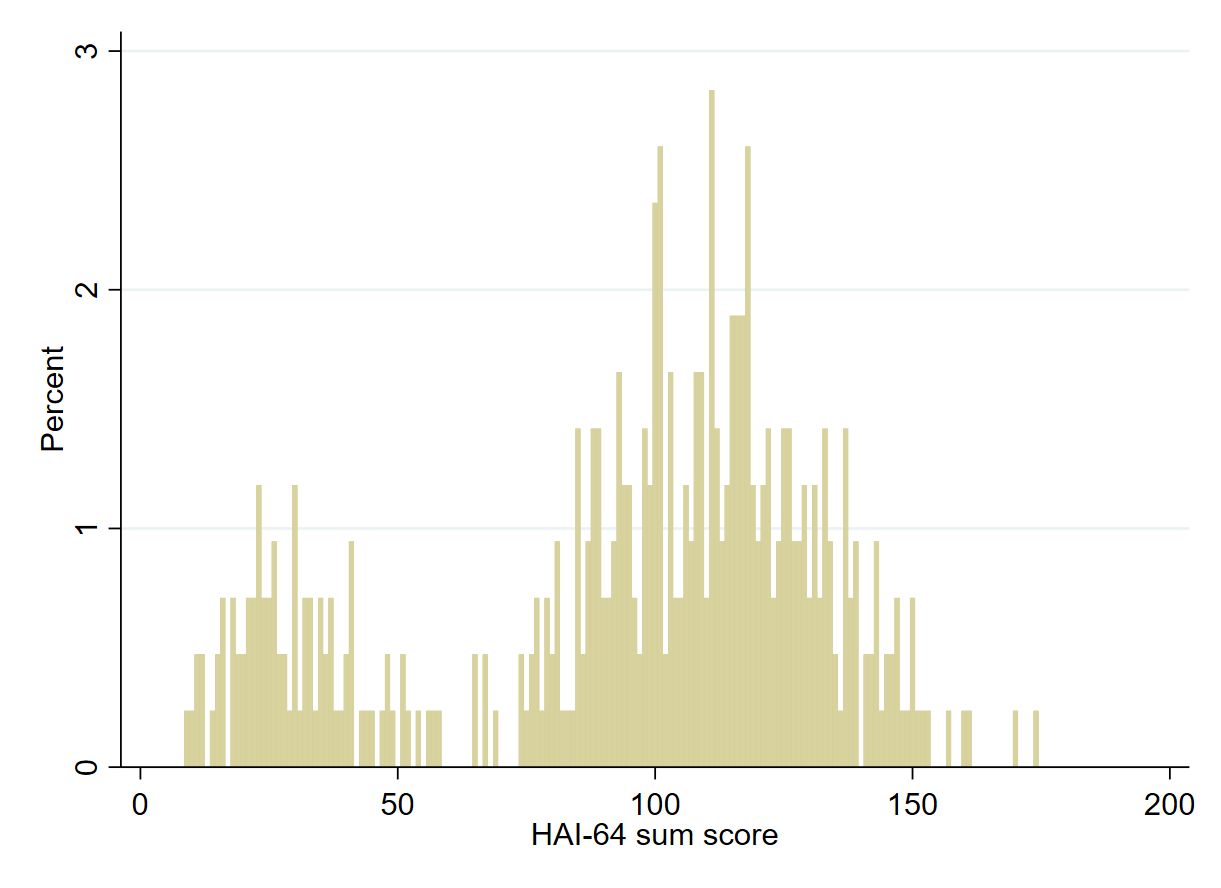


*Figure S3.* Histogram of 64-item Health Anxiety Inventory sum scores.

### *Sum score distribution of the 18-item Health Anxiety Inventory (HAI-18)*


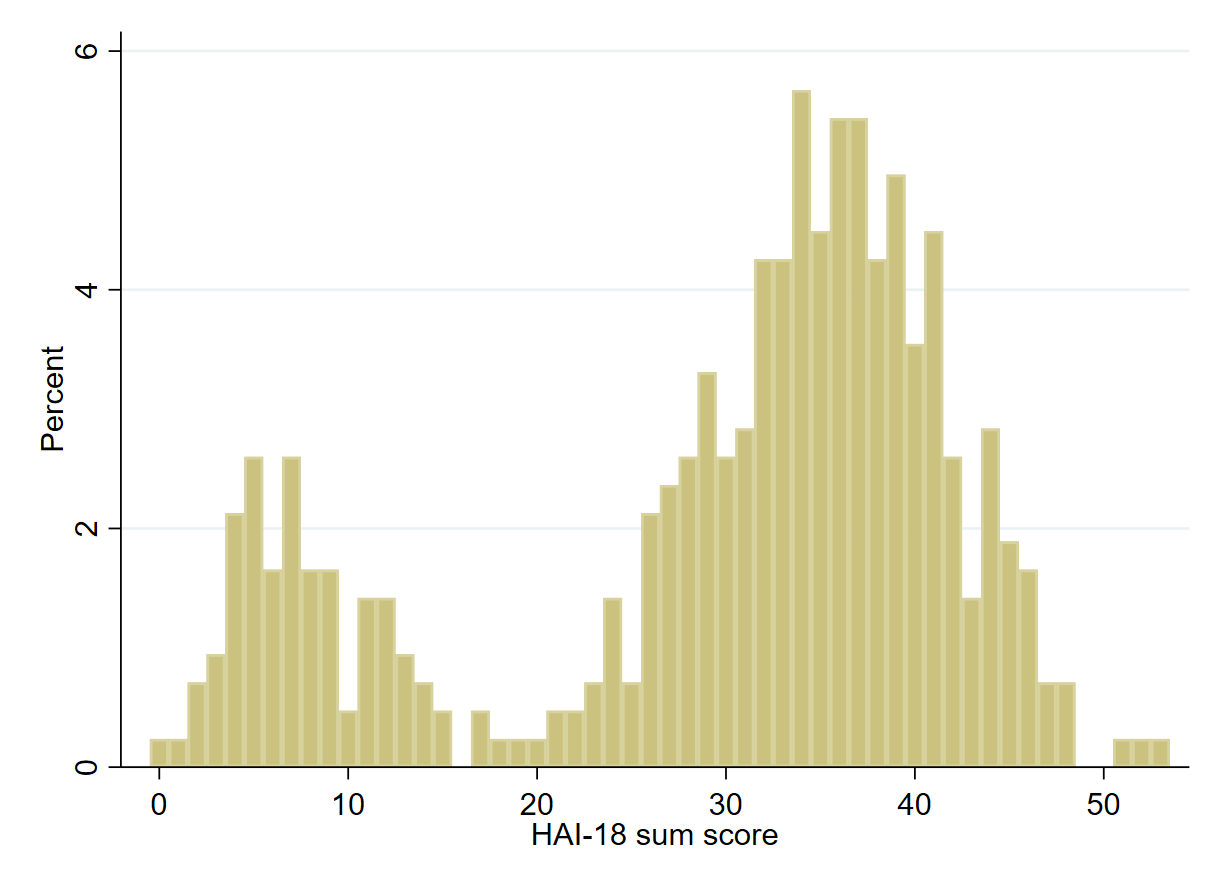


*Figure S4.* Histogram of 18-item Health Anxiety Inventory sum scores.

### *Sum score distribution of the 14-item Health Anxiety Inventory (HAI-14)*


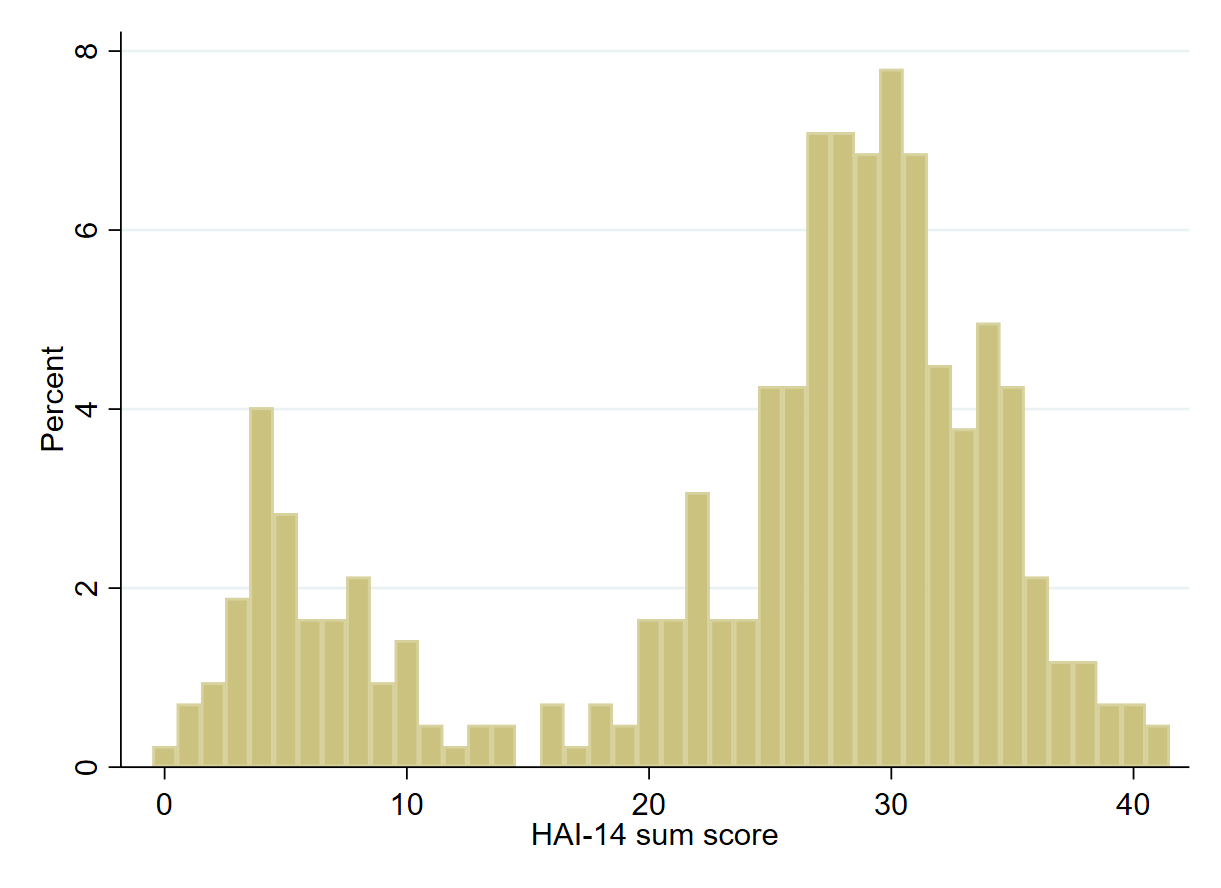


*Figure S5.* Histogram of 14-item Health Anxiety Inventory sum scores.

### *Sum score distribution of the Illness Attitude Scale (IAS)*


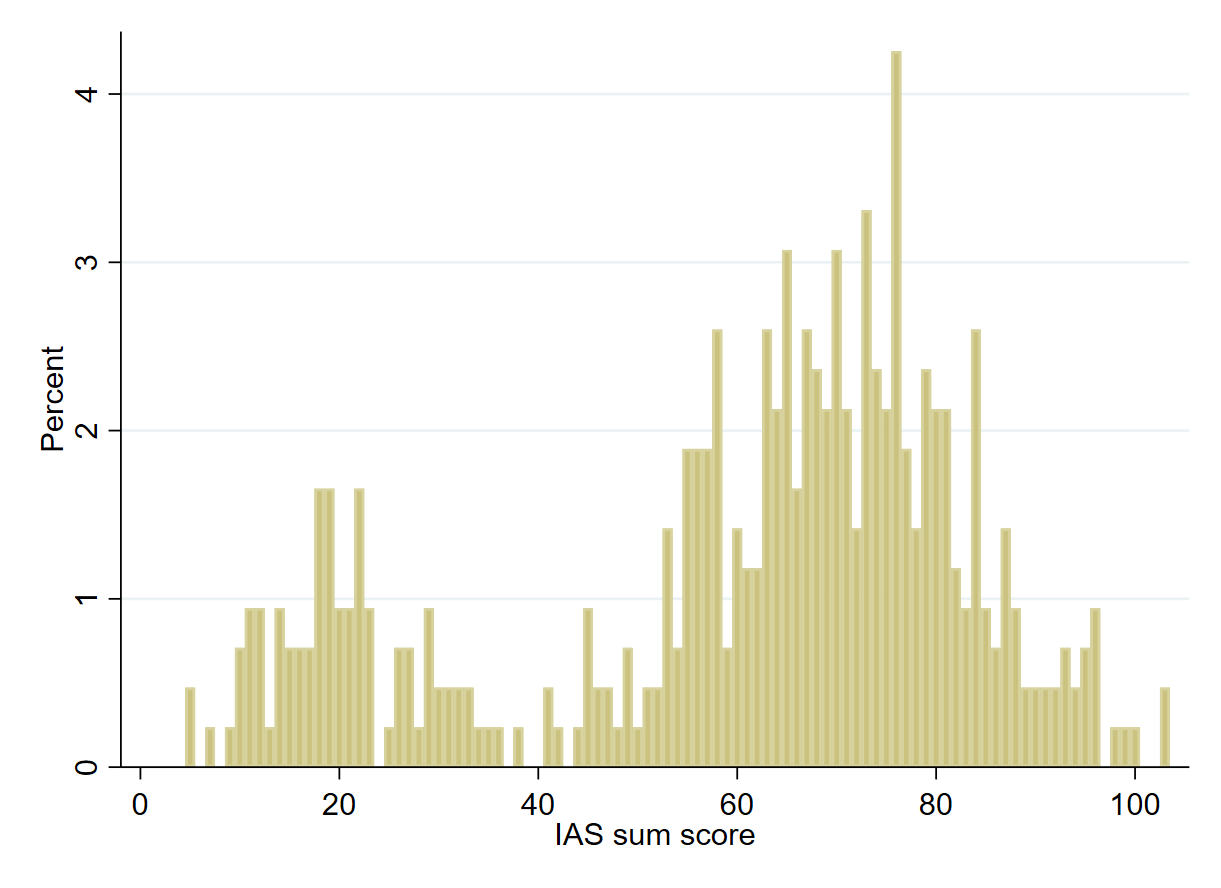


*Figure S6.* Histogram of Illness Attitude Scale sum scores.

### *Sum score distribution of the 14-item Whiteley Index with yes/no items (WI-14)*


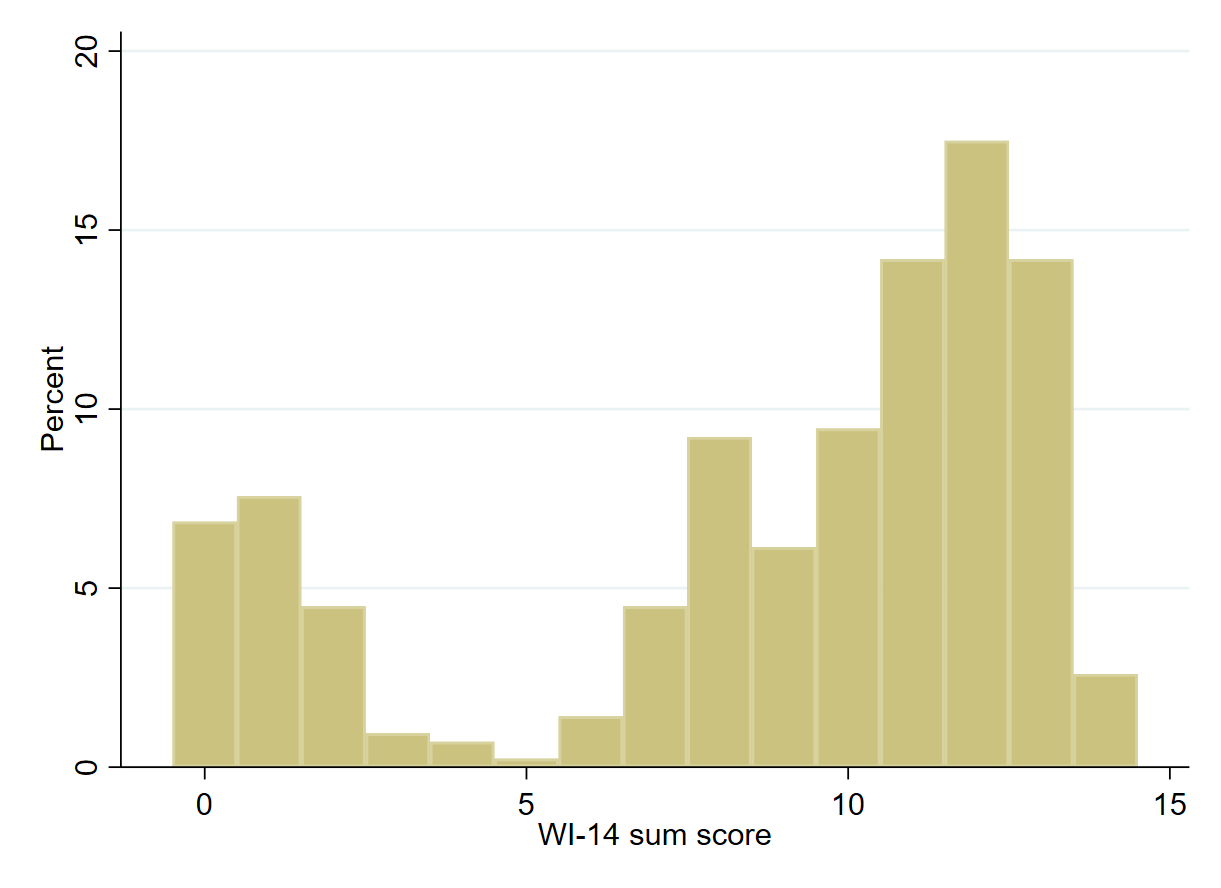


*Figure S7.* Histogram of 14-item Whiteley Index sum scores.
