# Supplementary material for: Joint factor analysis and approximate equipercentile linking of common trait health anxiety measures: a cross-sectional study of the 14-, 18- and 64-item health anxiety inventory, the illness attitude scale, and the 14-item Whiteley Index
Source: BMC Psychiatry. 2023 Sep 6;23:658. doi: 10.1186/s12888-023-05151-7 (PMC10483785; doi:10.1186/s12888-023-05151-7)
Supplement: Supplementary file 3 — Supplementary Material 3: Sensitivity analysis: alternative linking based on non-smoothed frequency distributions [file 12888_2023_5151_MOESM3_ESM.docx]

# Supplementary material:

# Sensitivity analysis: alternative linking based on non-smoothed frequency distributions

## Supplement of “Joint factor analysis and approximate equipercentile linking of common trait health anxiety measures: A cross-sectional study of the 14-, 18- and 64-item Health Anxiety Inventory, the Illness Attitude Scale, and the 14-item Whiteley Index”

*Table S7.* Sensitivity analysis: alternative cross-walk table of equipercentile linked scores on trait health anxiety measures as based on non-presmoothed frequency distributions

| Percentile | HAI-14 | HAI-18 | HAI-64 | IAS | WI-14 |  | Percentile | HAI-14 | HAI-18 | HAI-64 | IAS | WI-14 |
| --- | --- | --- | --- | --- | --- | --- | --- | --- | --- | --- | --- | --- |
| 0.00 | 0 | 0 | 9 |  |  |  | 39.25 |  | 31 | 96 |  |  |
| 0.25 |  | 1 | 10 | 5 |  |  | 39.50 |  |  |  | 62 |  |
| 0.50 | 1 |  |  | 7 |  |  | 40.00 |  |  | 97 |  |  |
| 0.75 |  | 2 | 11 | 9 |  |  | 41.00 |  |  | 98 |  |  |
| 1.25 |  |  | 12 | 10 |  |  | 41.50 |  |  |  | 63 |  |
| 1.50 | 2 |  | 14 |  |  |  | 42.25 |  |  | 99 |  |  |
| 1.75 |  | 3 |  |  |  |  | 42.75 |  | 32 |  |  |  |
| 2.00 |  |  | 15 |  |  |  | 43.75 |  |  |  | 64 |  |
| 2.25 |  |  |  | 11 |  |  | 44.00 | 27 |  | 100 |  |  |
| 2.50 |  |  | 16 |  |  |  | 46.50 |  |  | 101 | 65 |  |
| 2.75 | 3 |  |  |  |  |  | 46.75 |  |  |  |  | 10 |
| 3.00 |  |  |  | 12 |  |  | 47.00 |  | 33 |  |  |  |
| 3.25 |  | 4 | 18 |  |  |  | 48.00 |  |  | 102 |  |  |
| 3.50 |  |  |  |  | 0 |  | 48.75 |  |  |  | 66 |  |
| 3.75 |  |  | 19 | 13 |  |  | 49.00 |  |  | 103 |  |  |
| 4.25 |  |  | 20 | 14 |  |  | 50.25 |  |  | 104 |  |  |
| 4.75 |  |  | 21 |  |  |  | 51.00 | 28 |  | 105 | 67 |  |
| 5.00 |  |  |  | 15 |  |  | 52.00 |  | 34 | 106 |  |  |
| 5.50 |  | 5 | 22 |  |  |  | 53.00 |  |  | 107 |  |  |
| 5.75 | 4 |  |  | 16 |  |  | 53.50 |  |  |  | 68 |  |
| 6.50 |  |  | 23 | 17 |  |  | 54.25 |  |  | 108 |  |  |
| 7.50 |  |  | 24 |  |  |  | 55.75 |  |  |  | 69 |  |
| 7.75 |  | 6 |  | 18 |  |  | 56.00 |  |  | 109 |  |  |
| 8.25 |  |  | 25 |  |  |  | 57.00 |  | 35 | 110 |  |  |
| 9.00 |  |  | 26 |  |  |  | 58.00 | 29 |  |  |  |  |
| 9.25 | 5 |  |  | 19 |  |  | 58.25 |  |  |  | 70 |  |
| 9.75 |  | 7 | 27 |  |  |  | 58.75 |  |  | 111 |  | 11 |
| 10.25 |  |  | 28 |  |  |  | 60.75 |  |  |  | 71 |  |
| 10.50 |  |  | 29 |  |  |  | 61.00 |  |  | 112 |  |  |
| 10.75 |  |  |  | 20 | 1 |  | 62.00 |  | 36 |  |  |  |
| 11.25 |  |  | 30 |  |  |  | 62.25 |  |  | 113 |  |  |
| 11.50 | 6 |  |  | 21 |  |  | 62.75 |  |  |  | 72 |  |
| 12.00 |  | 8 | 31 |  |  |  | 63.25 |  |  | 114 |  |  |
| 12.50 |  |  | 32 |  |  |  | 64.75 |  |  | 115 |  |  |
| 13.00 | 7 |  | 33 | 22 |  |  | 65.00 |  |  |  | 73 |  |
| 13.50 |  | 9 | 34 |  |  |  | 65.25 | 30 |  |  |  |  |
| 14.00 |  |  | 35 |  |  |  | 66.75 |  |  | 116 |  |  |
| 14.25 |  |  |  | 23 |  |  | 67.50 |  | 37 |  |  |  |
| 14.75 |  | 10 | 36 | 25 |  |  | 67.75 |  |  |  | 74 |  |
| 15.00 | 8 |  |  |  |  |  | 68.50 |  |  | 117 |  |  |
| 15.25 |  |  | 37 | 26 |  |  | 70.00 |  |  |  | 75 |  |
| 15.50 |  | 11 |  |  |  |  | 70.75 |  |  | 118 |  |  |
| 15.75 |  |  | 38 |  |  |  | 72.25 |  | 38 |  |  |  |
| 16.00 |  |  | 39 | 27 |  |  | 72.75 | 31 |  | 119 |  |  |
| 16.25 |  |  | 40 |  |  |  | 73.25 |  |  |  | 76 |  |
| 16.50 | 9 |  |  | 28 |  |  | 73.75 |  |  | 120 |  |  |
| 16.75 |  |  |  |  | 2 |  | 74.50 |  |  |  |  | 12 |
| 17.00 |  | 12 | 41 | 29 |  |  | 74.75 |  |  | 121 |  |  |
| 17.50 |  |  | 43 |  |  |  | 76.00 |  |  | 122 |  |  |
| 17.75 | 10 |  | 44 | 30 |  |  | 76.25 |  |  |  | 77 |  |
| 18.00 |  |  | 45 |  |  |  | 77.00 |  | 39 |  |  |  |
| 18.25 |  | 13 | 47 | 31 |  |  | 77.25 |  |  | 123 |  |  |
| 18.75 | 11 |  | 48 | 32 |  |  | 78.00 |  |  | 124 | 78 |  |
| 19.00 | 12 | 14 | 49 |  |  |  | 78.25 | 32 |  |  |  |  |
| 19.25 |  |  |  | 33 |  |  | 79.25 |  |  | 125 |  |  |
| 19.50 | 13 | 15 | 51 | 34 | 3 |  | 80.00 |  |  |  | 79 |  |
| 19.75 | 14 |  | 52 | 35 |  |  | 80.50 |  |  | 126 |  |  |
| 20.00 |  | 17 | 54 | 36 |  |  | 81.25 |  | 40 |  |  |  |
| 20.25 |  |  | 56 | 38 | 4 |  | 81.75 |  |  | 127 |  |  |
| 20.50 | 16 | 18 | 57 | 41 |  |  | 82.25 |  |  |  | 80 |  |
| 20.75 |  | 19 | 58 |  | 5 |  | 82.50 | 33 |  |  |  |  |
| 21.00 | 17 | 20 | 65 | 42 |  |  | 82.75 |  |  | 128 |  |  |
| 21.25 |  | 21 |  | 44 |  |  | 83.75 |  |  | 129 |  |  |
| 21.50 | 18 |  | 67 |  | 6 |  | 84.25 |  |  |  | 81 |  |
| 21.75 |  | 22 | 69 | 45 |  |  | 84.75 |  |  | 130 |  |  |
| 22.00 | 19 |  |  |  |  |  | 85.25 |  | 41 |  |  |  |
| 22.25 |  | 23 | 74 |  |  |  | 85.75 |  |  | 131 |  |  |
| 22.50 |  |  | 75 | 46 |  |  | 86.00 |  |  |  | 82 |  |
| 23.00 | 20 |  | 76 | 47 |  |  | 86.75 |  |  | 132 |  |  |
| 23.25 |  |  |  | 48 |  |  | 87.00 | 34 |  |  | 83 |  |
| 23.50 |  | 24 | 77 |  |  |  | 87.75 |  |  | 133 |  |  |
| 23.75 |  |  |  | 49 |  |  | 88.75 |  | 42 |  | 84 |  |
| 24.00 |  |  | 78 |  |  |  | 89.00 |  |  | 134 |  |  |
| 24.25 |  |  |  | 50 |  |  | 89.50 |  |  | 135 |  |  |
| 24.50 |  | 25 | 79 | 51 | 7 |  | 90.00 |  |  | 136 |  |  |
| 24.75 | 21 |  |  |  |  |  | 90.25 |  |  |  |  | 13 |
| 25.00 |  |  | 80 | 52 |  |  | 90.50 |  |  |  | 85 |  |
| 25.75 |  |  | 81 |  |  |  | 90.75 |  | 43 | 137 |  |  |
| 26.00 |  | 26 |  | 53 |  |  | 91.25 |  |  |  | 86 |  |
| 26.25 |  |  | 82 |  |  |  | 91.50 | 35 |  |  |  |  |
| 26.50 |  |  | 83 |  |  |  | 91.75 |  |  | 138 |  |  |
| 26.75 |  |  | 84 |  |  |  | 92.50 |  |  |  | 87 |  |
| 27.00 | 22 |  |  | 54 |  |  | 92.75 |  |  | 139 |  |  |
| 27.75 |  |  | 85 |  |  |  | 93.00 |  | 44 |  |  |  |
| 28.25 |  | 27 |  | 55 |  |  | 93.50 |  |  | 141 | 88 |  |
| 28.50 |  |  | 86 |  |  |  | 93.75 |  |  | 142 |  |  |
| 29.25 |  |  | 87 |  |  |  | 94.25 |  |  |  | 89 |  |
| 29.50 | 23 |  |  |  |  |  | 94.50 |  |  | 143 |  |  |
| 30.25 |  |  |  | 56 |  |  | 94.75 | 36 |  |  | 90 |  |
| 30.50 |  | 28 | 88 |  |  |  | 95.25 |  | 45 | 144 | 91 |  |
| 31.00 | 24 |  |  |  |  |  | 95.50 |  |  | 145 |  |  |
| 31.25 |  |  |  |  | 8 |  | 95.75 |  |  |  | 92 |  |
| 32.00 |  |  | 89 |  |  |  | 96.00 |  |  | 146 |  |  |
| 32.25 |  |  |  | 57 |  |  | 96.25 | 37 |  |  | 93 |  |
| 33.00 |  |  | 90 |  |  |  | 96.50 |  |  | 147 |  |  |
| 33.50 |  | 29 |  |  |  |  | 97.00 |  | 46 | 148 | 94 |  |
| 33.75 |  |  | 91 |  |  |  | 97.25 |  |  | 149 |  |  |
| 34.00 | 25 |  |  |  |  |  | 97.50 | 38 |  |  | 95 |  |
| 34.50 |  |  | 92 | 58 |  |  | 97.75 |  |  | 150 |  |  |
| 35.75 |  |  | 93 |  |  |  | 98.25 |  | 47 | 151 | 96 |  |
| 36.00 |  |  |  | 59 |  |  | 98.50 | 39 |  | 152 |  |  |
| 36.50 |  | 30 |  |  |  |  | 98.75 |  |  | 153 |  | 14 |
| 37.00 |  |  |  | 60 |  |  | 99.00 |  | 48 | 157 | 98 |  |
| 37.25 |  |  | 94 |  |  |  | 99.25 | 40 |  | 160 | 99 |  |
| 38.25 | 26 |  |  |  |  |  | 99.50 |  | 51 | 161 | 100 |  |
| 38.50 |  |  | 95 | 61 |  |  | 99.75 | 41 | 52 | 170 | 103 |  |
| 39.00 |  |  |  |  | 9 |  | 100.00 |  | 53 | 174 |  |  |

*Note.* This is a sensitivity analysis based on non-presmoothed frequency distributions. Using this table, each trait health anxiety score observed in the present study can be linked to its percentile, which in turn can be linked to a score on another trait health anxiety scale. HAI = Health Anxiety Inventory (14, 18, and 64-item version as indicated); IAS = Illness Attitude Scale; WI-14 = 14-item Whiteley Index with dichotomous (“yes”/”no”) items.
